# Cell Volume Changes and Membrane Ruptures Induced by Hypotonic Electrolyte and Sugar Solutions

**DOI:** 10.3389/fphys.2020.582781

**Published:** 2020-12-07

**Authors:** Bojan Božič, Špela Zemljič Jokhadar, Luka Kristanc, Gregor Gomišček

**Affiliations:** ^1^Institute of Biophysics, Faculty of Medicine, University of Ljubljana, Ljubljana, Slovenia; ^2^Faculty of Health Sciences, University of Ljubljana, Ljubljana, Slovenia

**Keywords:** osmotic effects, CHO cells, hypotonic medium, cell volume, tension-induced rupture

## Abstract

The cell volume changes induced by hypotonic electrolyte and sucrose solutions were studied in Chinese-hamster-ovary epithelial cells. The effects in the solutions with osmolarities between 32 and 315 mosM/L and distilled water were analyzed using bright-field and fluorescence confocal microscopy. The changes of the cell volume, accompanied by the detachment of cells, the formation of blebs, and the occurrence of almost spherical vesicle-like cells (“cell-vesicles”), showed significant differences in the long-time responses of the cells in the electrolyte solutions compared with the sucrose-containing solutions. A theoretical model based on different permeabilities of ions and sucrose molecules and on the action of Na^+^/K^+^-ATPase pumps is applied. It is consistent with the observed temporal behavior of the cells’ volume and the occurrence of tension-induced membrane ruptures and explains lower long-time responses of the cells in the sucrose solutions.

## Introduction

Variations in cell volume have to be kept to a minimum in order to maintain the structural and functional integrity of the cell (McManus et al., 1995; Lang et al., 1998; Strange, 2004; Hohmann, 2015). This is of special importance for animal cells that have, compared with organisms with more-or-less elaborate rigid cell walls (e.g., bacteria, fungi, and plants), significantly lower mechanical stiffness of the membrane and thus a lower resistance to intracellular osmotic pressure buildup.

The cellular responses to the changes in the osmolarity of the cell’s surroundings have been widely observed (Hall et al., 1996; Lang et al., 1998; Reuss et al., 2004; Groulx et al., 2006; Kiesel et al., 2006; Usaj et al., 2009; Pietuch et al., 2013). Normally, the cell is able to adjust its cytosolic osmolarity to the surroundings by employing the transport mechanisms of water, electrolytes (Na^+^, K^+^, and Cl^–^ ions), and organic osmolytes (polyols, amino acids, and methylamines) (Lang et al., 1998). However, if the cell is exposed to the more severe and persistent osmolarity change, it preserves its integrity by additional mechanisms known by the expressions regulatory volume decrease (RVD) and regulatory volume increase (RVI) (Lang et al., 1998; Okada et al., 2001; Strange, 2004; Groulx et al., 2006). These mechanisms include the shape transformations and the flattening of the membrane invaginations (Echarri and Del Pozo, 2015; Echarri et al., 2019), the activation of additional membrane transport mechanisms (Jennings and Schulz, 1990; Sarkadi and Parker, 1991; Kirkegaard et al., 2016), and the upregulation of osmoregulatory proteins and molecular chaperonins to counteract the protein unfolding (Burg and Garcia-Perez, 1992; Caruccio et al., 1997). The channels that drastically increase the permeability of the cell membrane were believed to be ion-selective (Stutzin et al., 1999; Culliford et al., 2004); however, many studies indicate that smaller organic osmolytes can also pass through these channels (Jackson and Strange, 1993; Strange and Jackson, 1995; Hall et al., 1996). Many putative molecular triggers of the RVD have been revealed, e.g., a membrane tension increase, an alteration of the cytoskeletal architecture, or changes of the concentrations of specific cytoplasmic molecules or cellular ions, detected by mechanoreceptors or chemoreceptors (Duan et al., 1997; Okada et al., 2001; Strange, 2004; Qiu et al., 2014; Voss et al., 2014; de Los Heros et al., 2018; Orlov et al., 2018).

The complex volumetric response of the cells has been the subject of several theoretical studies (Reuss et al., 2004; Zemljic Jokhadar et al., 2016; Fazelkhah et al., 2018). The response of Jurkat cells to the hypotonic stress was modeled by the volume-sensitive channel (VSC) model (Reuss et al., 2004). Recently, a quantitative model describing the ion transport and the cytoplasm conductivity of the Chinese-hamster-ovary (CHO) epithelial cells was presented (Fazelkhah et al., 2018). CHO-specific model parameters, e.g., the cellular ion concentrations and permeabilities, were determined using a flux assay approach (Fazelkhah et al., 2018). A theoretical model, which includes also the occurrence of membrane ruptures (i.e., tension pores), was developed in order to understand the responses of the CHO epithelial cells induced by a pore-forming polyene nystatin (Zemljic Jokhadar et al., 2016). It is based on the theory of osmotic lysis (Koslov and Markin, 1984) and on the pore-formation theory (Huang et al., 2004). Namely, an increase of the cell volume occurs after the formation of the size-discriminating nystatin pores that can lead to critical membrane tension and, consequently, to membrane rupture. The characteristics of the tension-induced ruptures have been studied in phospholipid vesicles that represent a relatively simple model for the cell membranes (Bloom et al., 1991; Brochard-Wyart et al., 2000; Evans et al., 2003; Hsueh et al., 2007; Kristanc et al., 2014; Chabanon et al., 2017; Liviu and Dumitru, 2019). Their behavior has been explained by theoretical models based on the theory of osmotic lysis (Koslov and Markin, 1984). The experimental studies on phospholipid vesicles showed that the critical lateral membrane tension of the lipid bilayers, which is dependent on the lipid composition and the rate of tension change, is in the range between 1 and 25 mN/m (Bloom et al., 1991; Evans et al., 2003). This leaves only a limited space of approximately 3% membrane area for its expansion (Nichol and Hutter, 1996; Hamill and Martinac, 2001), which can be determined by the ratio between the critical membrane tension (σ_*C*_) and membrane-stretching constant (*k*_*A*_). This expansion corresponds to a 5% cell volume increase of the spherical vesicle in giant unilamellar vesicles. Different types of tension-induced ruptures, such as transient ruptures (lasting around 100 ms) and longer lasting vesicle ruptures (order of magnitude 1 s and longer), which depend on the osmolarity difference across the vesicle’s membrane and its temporal evolvement, were predicted and determined (Brochard-Wyart et al., 2000; Mally et al., 2007; Kristanc et al., 2014; Chabanon et al., 2017; Liviu and Dumitru, 2019).

Our aim was to study the effects induced in mammalian cells exposed to hypotonic solutions of electrolytes, i.e., Leibovitz-water solutions, and sugar solutions, i.e., sucrose-water solutions. We focused on the characteristic volume changes and on the behavior of tension-induced ruptures in CHO cells. A theoretical model that was primarily applied for the description of the osmotic phenomena induced by the pore-forming agent was upgraded by considering different permeabilities of ions and the action of Na^+^/K^+^-ATPase pumps. Significant differences in the long-time volume responses of the CHO cells and in the occurrence of tension-induced ruptures were observed in the electrolyte and sugar solutions. Two main questions addressed in this study are: (i) can the model presented describe the complex volumetric behavior of the cell and, especially, the occurrence of tension-induced ruptures at extreme hypotonicity? and (ii) can the observed differences in the electrolyte and sugar solutions be predicted and explained by the model?

## Materials and Methods

### Preparation of the CHO Cells

The CHO epithelial cells (ECAC) were grown in a minimum essential medium (Gibco, United States) (growth medium) at 37°C in a CO_2_ incubator (Kambic, Slo). CHO cells were seeded (20,000 cells/ml) in a self-fabricated dish [polydimethylsiloxane (PDMS) chamber] with a glass slide bottom 1 day prior to the experiments.

Just before the time-lapse imaging with the bright-field illumination was started, the PDMS chamber with the cells was transferred to the microscope stage, and the growth medium was substituted with the hypotonic solutions of interest. For leakage experiments, the calcein AM solution (Life Technologies, United States) was added to the cells in the growth medium 20 min before the measurement. For the observations of the cellular morphological changes and the cells’ volume determination, the CellMask plasma membrane stain (Life Technologies, United States) was added to the cells for the same period. After the incubation, the cells were carefully washed and transferred to the microscope, where the growth medium was substituted with the hypotonic solutions of interest just before the imaging was started. In all imaging experiments, the cells were heated to ensure a constant temperature of 37°C. The pH values were kept in a physiological range. A more detailed description can be found elsewhere (Zemljic Jokhadar et al., 2016).

The contribution of the Na^+^/K^+^-ATPase pumps was evaluated by partially repeating the experiments in the presence of ouabain that is known to reduce the active ion transport (Strange, 1989). The cells were pretreated by ouabain at a concentration of 1 μmol/L for 24 h. Afterward, they were exposed to the Leibovitz’s solution that was diluted by 60% with distilled water and to the sucrose solution with a comparable osmolarity (126 mosM/L). The cells were monitored for 60 min, and their volumes were determined as in other experiments in the study. Both solutions included ouabain at the same concentration.

### Experimental Set-Up and Procedure

The cells were studied with bright-field and fluorescence microscopy using a Nikon ECLIPSE TE2000-E microscope (Plan Apo TIRF objective, magnification 60×, NA = 1.45). Using the bright-field technique, the images were captured with a digital camera (DS-2M BW; Nikon, Japan). For confocal microscopy, the Nikon C1 system was used (light source: xenon–argon laser 488, excitation filter: EX510-560, absorption filter: BA590). The images were recorded within the same field of view for 60 min at a rate of 1 frame/s in the bright-field mode and at a rate from 1 frame/s to 1 frame/10 min in the fluorescence microscopy.

The hypotonic solutions were obtained by either (i) the addition of distilled water (J. T. Baker, Poland) to the Leibovitz’s L-15 medium (Gibco, United States) supplemented with 10% fetal bovine serum (FBS) (Leibovitz’s medium) or (ii) the preparation of different sucrose-water solutions (Sigma, United States). In the first case, the Leibovitz’s medium was diluted by 90, 80, 60, 40, or 20% with distilled water, leading to Leibovitz-water solutions with osmolarities equal to 32, 63, 126, 189, and 252 mosM/L. In the second case, different sucrose-water solutions whose osmolarities corresponded to those of the Leibovitz-water solutions were prepared. Finally, the cells were also measured in the distilled water.

### Analysis of the Images

Qualitatively, the characteristic cell behavior and the leakage of the cell content were determined from the observations of bright-field and fluorescence images by three independent observers.

**FIGURE 1:**
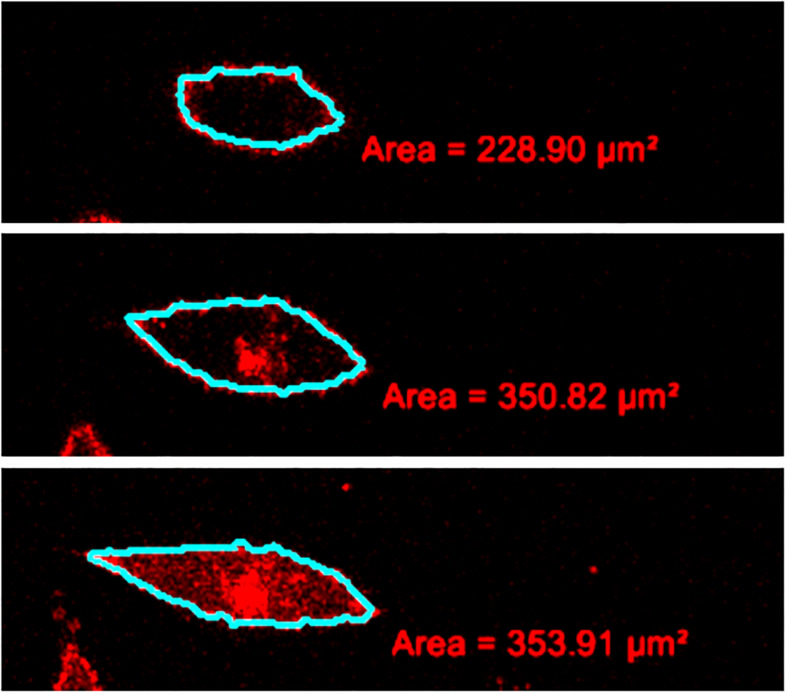
Example of a measurement of the cell volume from the stack of confocal images. Some consecutive slices with the drawn outlines of the cell membrane are depicted from the bottom of the cell to the top (from below upward).

Quantitatively, the cell volume was determined by the addition of the volumes of consecutive cell slices obtained from the confocal microscopy (Zemljic Jokhadar et al., 2016). For the volume determination of each slice in the stack, the cell cross-section was manually delineated from its surroundings, and the obtained cross-section area was multiplied by the step size (Figure 1). The relative standard deviations of the measurement of relative cell volume due to different observers were estimated to be less than 5%.

For the evaluation of the experimental data, the regular statistic procedures including the average values, medians, errors of the mean value, and standard deviations of the values normalized relative to their initial values were employed. The significance analysis was performed following Student’s *t* distribution. The two mean values of sets of data were considered to be significantly different with the probability value (*p*-value) lower than 0.05.

### Control Measurements

The CHO cells were measured firstly in Leibovitz’s medium without any addition of water and, secondly, in a sucrose-water solution with an osmolarity equal to 315 mosM/L, which corresponded to the osmolarity of Leibovitz’s medium. The cells in the control experiments were treated and analyzed in the same way as the cells in the hypotonic media.

### Viability Test

The cell viability was determined using the MTS test. The cells were plated in 96-well microtiter plates (100 μL; TPP, Switzerland) at a concentration of 5,000 cells/well 1 day before the treatment for 24 h in the growth medium and then treated for 60 min in different hypotonic Leibovitz-water or sucrose-water solutions. Afterward, the hypotonic solution of interest was replaced by fresh growth medium in the cell culture for another 48 h, followed by the addition of MTS reagent (CellTiter 96 AQueous Reagent; Promega, United States) for 1 h. In the control measurements, the cells were treated for 60 min in either undiluted Leibovitz’s medium or an iso-osmolar sucrose-water solution. The absorbance, which corresponds to the amount of formed soluble formazan product that is directly proportional to the number of viable cells, was measured at a wavelength of 490 nm. The cell viability was determined as the ratio of the absorbance measured in the cells in different hypotonic solutions and the absorbance in the undiluted Leibovitz’s medium.

## Experimental Results

### Results Obtained in the Diluted Leibovitz’s Medium

**FIGURE 2:**
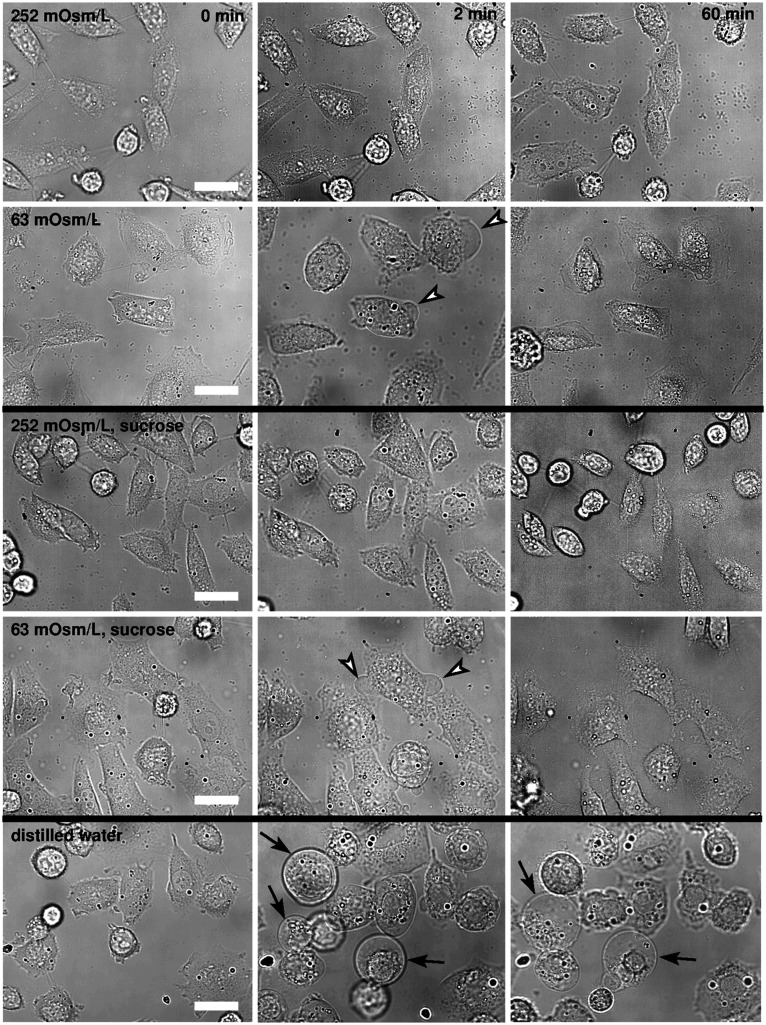
Bright-field images of CHO cells in hypotonic media. The cells are presented in the Leibovitz-water solutions with 252 and 63 mosM/L (first and second rows), in the sucrose-water solutions of the same osmolarities as the Leibovitz-water solutions (third and fourth rows), and in the distilled water (fifth row). The left-hand-side images present the cells before the hypotonic medium was added, the middle ones after 2 min, and the right-hand-side images 1 h after the addition of the diluted medium. Representative blebs are denoted by the arrowheads in the second and in the fourth row, and the “cell-vesicles” are denoted by the arrows in the fifth row, respectively. Images are presented in the same field of view, and the white bars represent 10 μm.

In the Leibovitz-water solution with the osmolarity of 252 mosM/L, no obvious cell-shape changes were detected during the observation time of 1 h (Figure 2), which is similar to the results obtained in the undiluted Leibovitz’s medium.

In the Leibovitz-water solutions with osmolarities equal to 189, 126, and 63 mosM/L, cells swelled instantaneously. The major shape changes, including the formation of smaller and bigger blebs, occurred within the first minute after the exposure (Figure 2). The blebs retracted back into the cell’s body in the first 5 min after their exposure, and only minor differences in the shape could be observed afterward between the control and treated cells using bright-field microscopy. The cell detachment on its periphery was not observed, and the cell footprint looked alike.

**FIGURE 3:**
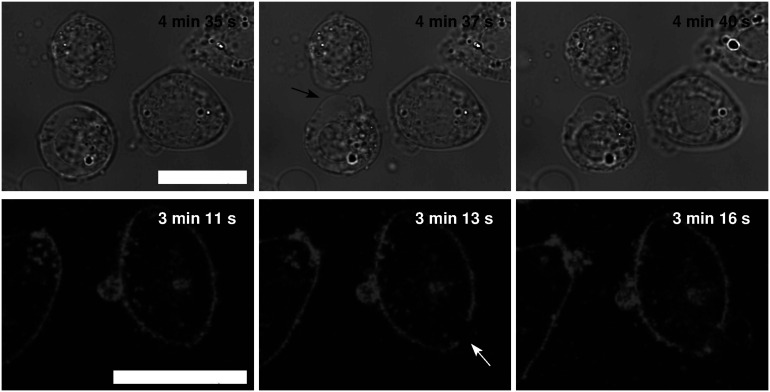
Slow “cell-vesicle” ruptures as observed by the bright-field and fluorescence microscopy. The images on the left-hand side represent “cell-vesicle” right before the slow rupture, the middle ones during, and the ones on the right-hand side after the rupture (bright-field upper and fluorescence lower rows). The tension pores are denoted by the arrows. The times of the cell exposure to the distilled water are given in the images. The images in the same row are presented in the same field of view, and the white bars represent 10 μm.

In the Leibovitz-water solution with osmolarities equal to or lower than 32 mosM/L, larger blebs appeared and disappeared in different regions of the cell membrane. The neighboring blebs often merged, and single blebs with almost spherical shape, referred to as “cell-vesicles,” were formed for the first time (Figure 2, fifth row). The binding of the membrane to the cytoskeleton was disrupted, the attachment of the membrane to the substrate was minimized, and the cell started to behave like a strained lipid vesicle. Some slow ruptures, characterized by a significant leakage of the vesicle content and a substantial decrease of the “cell-vesicle” volume in a few seconds, were detected. In most cases, a “cell-vesicle” with a smaller radius was observed afterward (Figure 3 and Supplementary Video 1). In the distilled water, corresponding to the maximum osmolarity difference (gradient) between the cell interior and the exterior, the cells swelled instantaneously and formed “cell-vesicles” (Figure 2). Slow “cell-vesicle” ruptures were often observed.

**FIGURE 4:**
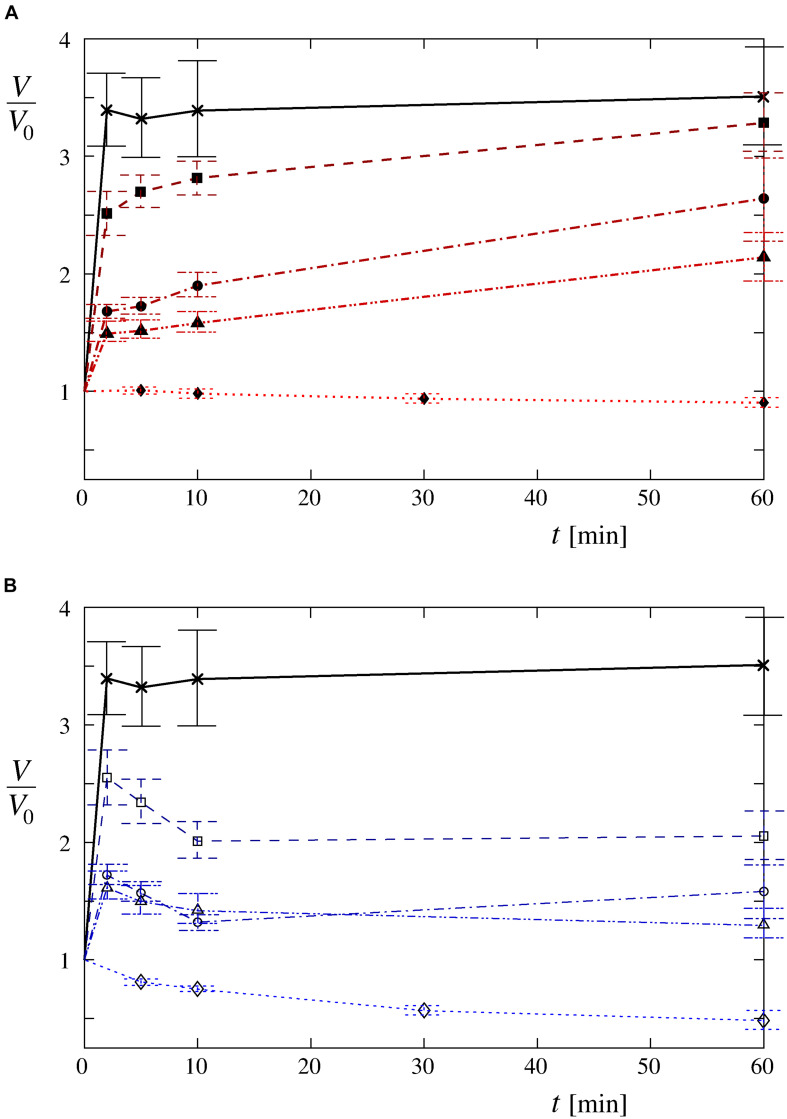
Time-behavior patterns of the average cell volume in hypotonic media. The average volume changes are plotted relative to their initial values for the cells exposed to **(A)** the Leibovitz-water solutions with the osmolarities of 189 (▲), 126 (●), and 63 mosM/L (■) and **(B)** the sucrose-water solutions with the same osmolarities [189 (Δ), 126 (o), and 63 mosM/L (□)]. The average volume changes for the cells in the distilled water (×) and for the control measurements in **(A)** undiluted Leibovitz’s medium (◆) and **(B)** in iso-osmolar sucrose solution (◆) are depicted as well. The lines are drawn to guide the eye. The number of cells in each group equals 12, which were determined in four or five independent experiments. For clarity, the standard deviations are not drawn in this figure, they are provided in Table 2. The standard errors of the mean value are drawn.

**FIGURE 5:**
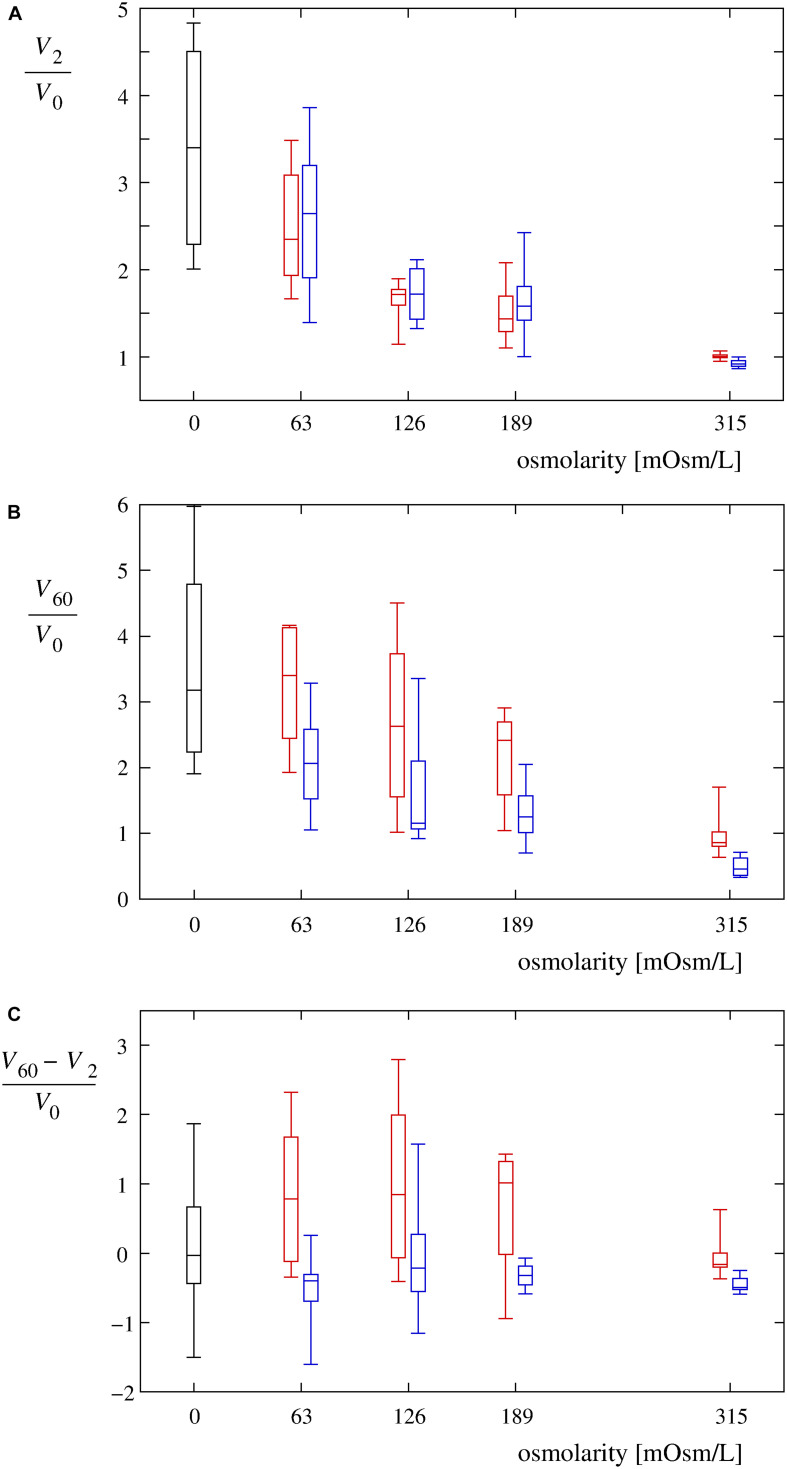
Volume changes of single cells in hypotonic media. **(A)** Normalized single-cell volumes (*V*_2_/*V*_0_) 2 min after the exposure to Leibovitz-water solutions (red) are drawn as a function of their osmolarities. Normalized single-cell volumes (*V*_2_/*V*_0_) 2 min after the exposure to sucrose-water solutions (blue) are depicted for the same osmolarities. The control values for the cells in an undiluted Leibovitz’s medium (red) and in an iso-osmolar sucrose solution (blue) are also shown. The volume changes of the cells exposed to the distilled water are shown in black. The single-cell volumes are normalized relative to their initial volumes. In each case, the boxes span over 50% of the measurements from the mean, the whiskers enclose the whole range of measurements, and the horizontal lines indicate the medians. Normalized single-cell volumes (*V*_60_/*V*_0_) 60 min after the exposure to different solutions and normalized differences between the single-cell volumes [(*V*_60_ – *V*_2_)/*V*_0_] 60 and 2 min after the exposure to different solutions of Leibovitz-water (red) and sucrose-water (blue) media with the corresponding osmolarities are drawn in **(B,C)**. The results of the control measurements in the undiluted Leibovitz’s medium (red) and in the iso-osmolar sucrose solution (blue) as well as of the cells exposed to the distilled water (black) are also shown. The number of cells in each group equals 12.

The volume changes, determined by the confocal microscopy, confirmed the dependency on the osmolarity difference between the cell interior and its surroundings. Faster increases of the cell volume in the first 2 min after the exposure of the cells to the hypotonic medium as well as higher maximum values reached during the whole observation period could clearly be seen at lower osmolarities of the Leibovitz-water solutions (Figures 4A, 5A). An average volume increase of 50% was observed in the first 2 min after the cell exposure to the hypotonic solution with an osmolarity of 189 mosM/L, whereas 70 and 150% volume increases were detected at osmolarities of 126 and 63 mosM/L, respectively (Figure 4A). An average volume increase as high as 240% was determined in the distilled water in the first 2 min. During the whole observation period, the average maximum relative volume increases were 120, 170, and 230% at osmolarities of 189, 126, and 63 mosM/L, respectively (Figure 4A). A maximum relative volume increase equal to approximately 250% was assessed in the distilled water.

**FIGURE 6:**
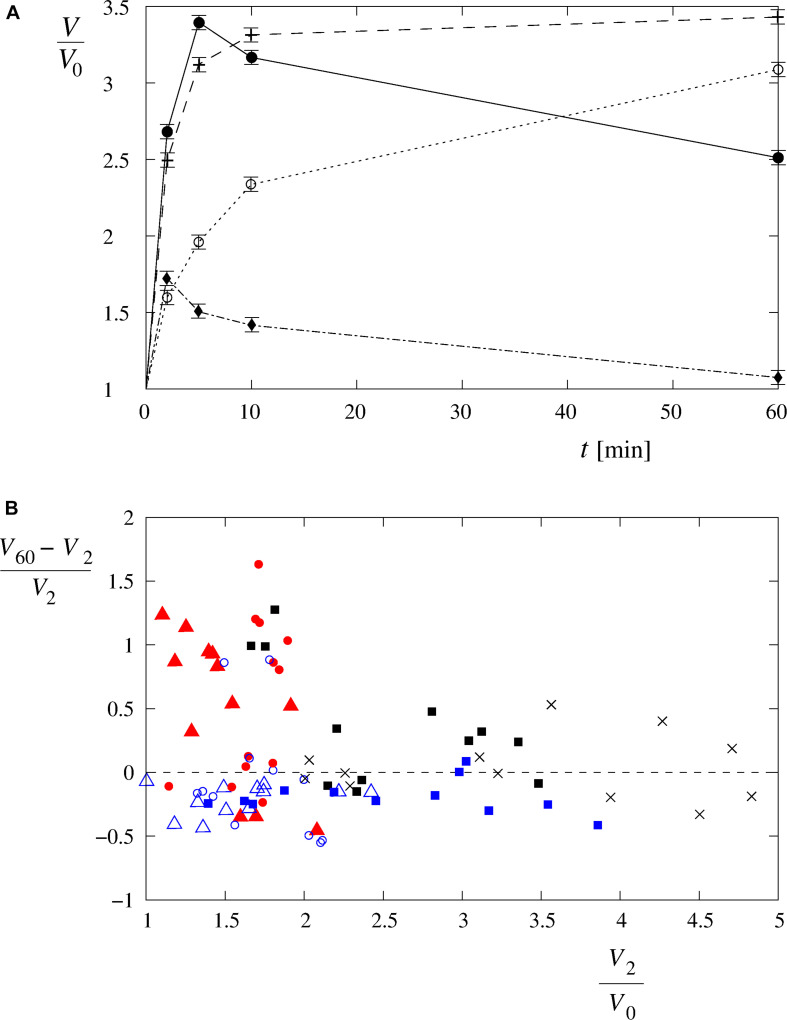
Four characteristic volume changes of the single cells exposed to hypotonic solutions. **(A)** The changes to the single-cell volumes can be characterized as “type A” (○), “type B” (+), “type C” (●), and “type D” (◆) time-behavior patterns. The choice is based on a total of 84 single-cell volume measurements in Leibovitz-water (*n* = 36) and in the sucrose-water solutions (*n* = 36) as well as in the distilled water (*n* = 12). The lines are drawn to guide the eye. **(B)** Distribution of the cell volume behavior as a function of (*V*_60_ – *V*_2_)/*V*_0_ and *V*_2_/*V*_0_. The symbols are described in Figure 5. Type A cell behavior [i.e., positive (*V*_60_ – *V*_2_)/*V*_0_, low *V*_2_/*V*_0_] lies on the left side above the dashed line, and type B with a fast increase at the beginning, followed by a fairly constant volume afterward [i.e., (*V*_60_ – *V*_2_)/*V*_0_ around 0, higher *V*_2_/*V*_0_], lies around the dashed line on the middle and right sides of the diagram. Type C with a fast increase at the beginning, followed by a volume decrease afterward [i.e., negative (*V*_60_ – *V*_2_)/*V*_0_, high *V*_2_/*V*_0_], lies below the dashed line on the right side of the diagram, and type D with a modest increase at the beginning, followed by a volume decrease afterward [i.e., negative (*V*_60_ – *V*_2_)/*V*_0_, low *V*_2_/*V*_0_], lies on the left side below the dashed line.

It should also be noted that four characteristic time-behavior patterns of the single-cell volume changes could be detected (Figure 6A): “type A” is characterized by a gradual, asymptotic increase in the cell volume during the entire measuring period; “type B” is characterized by a fast increase in the cell volume that attains its maximum plateau value shortly after the beginning of the measurement; “type C” is characterized by a rapid volume increase up to the critical value, followed by a gradual reduction of the cell volume; and “type D” is characterized by a volume increase up to its maximum value (lower than the critical value), followed by a gradual reduction of the cell volume. As seen in Figure 6B, the “type A” and “type D” cell behavior patterns were observed predominantly for osmolarities with values not lower than 63 mosM/L [“type A”: 61% in Leibovitz-water (*n* = 36) and 6% in sucrose-water (*n* = 36) solutions and “type D”: 14% in Leibovitz-water (*n* = 36) and 69% in sucrose-water (*n* = 36) solutions]. The “type B” and “type C” behavior patterns were more frequently observed in the experiments with osmolarities below 63 mosM/L and, in particular, in the distilled water [58% “type B” and 25% “type C” in distilled water (*n* = 12)].

### Control Measurements

The control experiments of the cells in the undiluted Leibovitz’s medium showed no significant changes to the cell shape during the whole observation time. On average, a continuous decrease in the cell volume to approximately 90% of its initial value was determined by quantitative measurements (Figures 4A, 5). In contrast, significant cell shrinkage was observed in the sucrose-water solution, whose osmolarity corresponded to the undiluted Leibovitz’s medium (315 mosM/L). In this case, the cell volume decreases to approximately 50% of its initial volume as determined (Figures 4B, 5). The volume decrease was more pronounced at the beginning of the measurement.

### Results Obtained in the Sucrose Solutions

The responses obtained by the bright-field microscopy in the cells that were exposed to the sucrose-water solutions with the osmolarities of 252, 189, 126, 63, and 32 mosM/L only partially correspond to those in the Leibovitz-water solutions. In the sucrose-water solution with an osmolarity equal to 252 mosM/L, no significant changes to the cell shape were detected during 1 h of the observation time, which is comparable to the cells in the undiluted Leibovitz’s medium (Figure 2). In the sucrose-water solutions with osmolarities equal to 189, 126, and 63 mosM/L, the cells, as seen with the bright-field microscopy, behaved in a similar way to the cells in the corresponding Leibovitz-water solutions. However, the swelling and the bleb-forming processes were less pronounced at the corresponding osmolarities.

In the sucrose-water solution with an osmolarity equal to 32 mosM/L, larger blebs compared with the sucrose-water solutions at higher osmolarities appeared in different regions of the cell membrane. However, no “cell-vesicles” were detected at this osmolarity. The shape changes were comparable to those seen in the cells exposed to the Leibovitz-water solution with an osmolarity equal to 63 mosM/L.

The cell volumes, determined by the confocal microscopy, showed comparable increases to those obtained in the corresponding Leibovitz-water solutions in the first 2 min after the cell exposure to the sucrose-water solutions (Figures 4, 5A). An average volume increase of 65% was detected in the first 2 min after the cell exposure to the sucrose-water solution with an osmolarity equal to 189 mosM/L, whereas 70 and 160% volume increases were obtained at 126 and 63 mosM/L, respectively (Figure 4B). Insignificant differences in the volume increases among the Leibovitz-water and sucrose-water solutions at these three osmolarities are also confirmed by *p-*values to be considerably higher than 0.05, with minimum *p-*value equal to 0.41. In contrast, significant differences among these solutions were observed at later times. The average volume increases reached their maximum values in the first 2 min after the cell exposure and started to decrease afterward (Figure 4B), which is also clearly reflected in the characteristic volume behavior patterns. They can be, in contrast to the cells exposed to the Leibovitz-water solutions, characterized predominantly (25 cases out of 36) by the “type D” behavior pattern (Figure 6). This is also exhibited by mostly negative differences between the single-cell volumes 60 and 2 min after their exposure to sucrose-water solutions (Figure 5C), which lead to highly significant differences (*p* < 10^–5^) when compared with the results of the corresponding Leibovitz-water solutions.

### Results Obtained With Ouabain Treatment

The exposure of the cells pretreated for 24 h with ouabain causes a comparable increase of their volumes in the Leibovitz’s medium diluted by 60% with distilled water and sucrose solution with corresponding osmolarity in the first minutes. An average cell volume increase of 1.55 (*SD* = 0.29) was determined in the diluted Leibovitz’s and 1.54 (*SD* = 0.17) in the sucrose solutions in the first 2 min. Afterward, the average cell volume of the ouabain-treated cells decreased only slightly in the diluted Leibovitz’s solution. In contrast, a pronounced decrease of the average cell volume was observed in the sucrose solution leading to highly significant differences in the cell volumes at longer times in the diluted ionic solution compared with the sucrose solution (*p* < 10^–3^). After 1 h of the observation time, for example, the average cell volume of the cells was only 6% smaller than the maximum cell volume in the diluted Leibovitz’s solution, whereas the corresponding cell volume exposed to the sucrose solution was diminished by 49%. The average cell volumes decreased to 1.44 (*SD* = 0.28) relative to the initial volume in the diluted Leibovitz’s solution and to 0.79 (*SD* = 0.13) in the corresponding sucrose solution. The number of cells in each group equals 12, which were determined in four independent experiments for each solution.

### Results Obtained in Calcein AM Marked Cells

**FIGURE 7:**
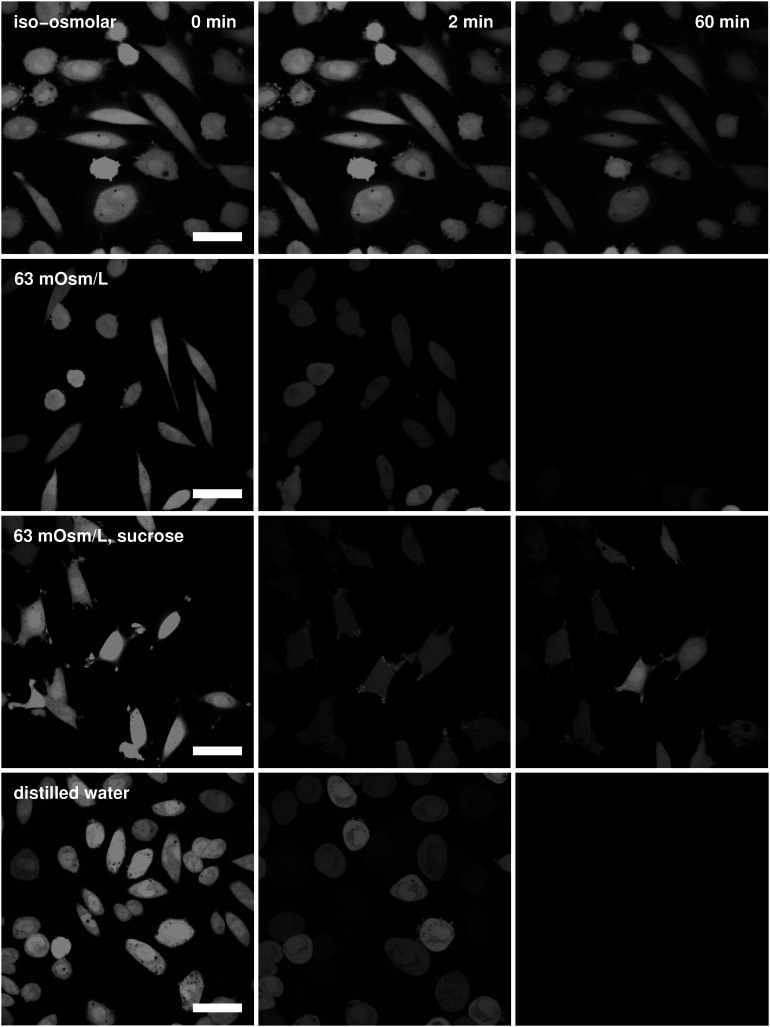
Fluorescent images of calcein-loaded cells. Images of the cells in the undiluted Leibovitz’s medium (first row), in the Leibovitz-water, and in the sucrose-water solutions with osmolarities of 63 mosM/L (second and third rows) and in the distilled water (fourth row) are shown for different time points (from left to right): before the exposure of the cells to the hypotonic medium, 2 and 60 min after their exposure. Images in each row are presented in the same field of view, whereas the white bars represent 20 μm.

The cells marked with the fluorescent calcein that were exposed to the undiluted Leibovitz’s medium showed no signal loss due to the calcein leakage during the whole observation time (Figure 7, first row). When the osmolarity of the Leibovitz-water solutions was decreased to 189, 126, or 63 mosM/L, a decrease in the fluorescent signal due to the calcein leakage was observed. On average, the signal started to decrease between 30 and 50 min after the exposure to the hypotonic medium, and it was not entirely lost in all the cells at the end of the measuring period (Figure 7, second row). In contrast, the cells started to lose their signal already in the first minute after their exposure to the Leibovitz-water solutions with an osmolarity equal to 32 mosM/L and to the distilled water (Figure 7, fourth row), where the largest signal drop normally occurred within 5 s. In general, an earlier and faster signal loss was more frequently encountered in more diluted solutions and especially in the distilled water.

On the other hand, the disappearance of the fluorescent signal due to the calcein leakage was rarely observed in the cells exposed to the sucrose-water solutions at osmolarities above or equal to 63 mosM/L during the whole observation time (Figure 7, third row). No signal loss was also encountered in the fluorescently marked cells in the control measurements. However, for osmolarities below 63 mosM/L, the signal loss could only be observed within the first 5 min after the exposure to the sucrose-water solutions. Fluorescent signal losses due to the calcein leakage were not observed after this initial period.

### Cell Viability

**FIGURE 8:**
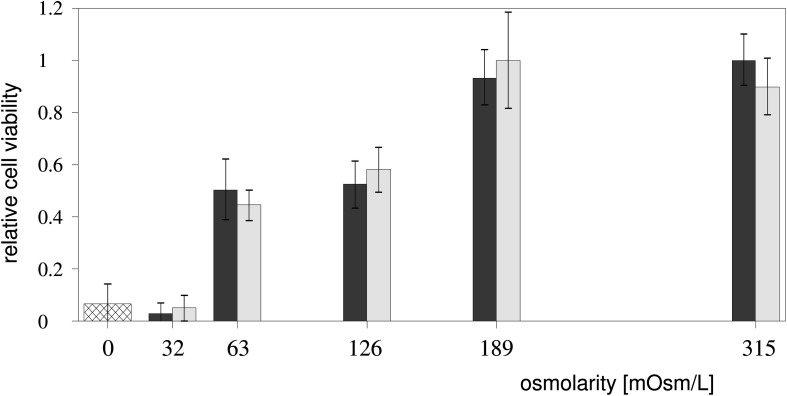
Cell viability in the hypotonic media. The percentage of viable cells (Viability Test section) depending on the exposure to the Leibovitz-water (gray) and to the sucrose-water (white) solutions with different osmolarities and to distilled water (cross-hatched) is shown. The results were obtained from three independent experiments. The viabilities, normalized to the undiluted Leibovitz’s medium, are presented as mean ± SD.

The cell viability, expressed as the percentage of viable cells according to the MTS test (Viability Test section), was found to be 93% in the hypotonic Leibovitz-water solution with an osmolarity of 189 mosM/L (Figure 8), which is significantly lower (*p* < 0.05) than the control value. The cell viability in the Leibovitz-water solutions with osmolarities of 126 and 63 mosM/L experienced a highly significant drop to values of around 50% (*p* < 10^–4^). The viability was found to be below 5% in the Leibovitz-water solution with an osmolarity equal to 32 mosM/L, which is comparable to the viability value found in distilled water.

The viability of the cells exposed to the sucrose-water solutions shows a comparable dependency on the osmolarities, as in the Leibovitz-water solutions (Figure 8).

## Theoretical Description

The primary goal of this section is to understand and to predict the behavior of the cells exposed to a hypotonic medium. For this purpose, a model describing the volume changes and the tension pore (tension-induced membrane rupture) behavior in cells, induced by the formation of transmembrane channels (Zemljic Jokhadar et al., 2016), was upgraded by considering the quantitative model for ion transport and the Na^+^/K^+^-ATPase pumps (Hernandez and Chifflet, 2000; Fazelkhah et al., 2018).

### The Model

#### Flow of Water

The volume of the cell in a hypotonic medium is increasing due to the inflow of water induced by a higher osmolarity inside the cell. The water flow through the cell membrane, which is driven by the differences in the osmotic pressure and in the hydrostatic pressure (Δ*p*) between the interior and the exterior of the cell, can be expressed as:

(1) $$J_{\text{m}}=Al_{\text{m}}\left(k_{\text{B}}T\sum_{i}\text{Δ}c_{i}-\Delta p\right)$$

where *A* is the membrane area, *l*_*m*_ is the permeability coefficient of the CHO cell membrane with respect to water, *k*_*B*_ is the Boltzmann constant, and *T* is the temperature. Δ*c*_*i*_ = *N*_*i*_/*V* - *c*_*i*_ is the difference of the solute concentrations inside and outside the cell, with *i* referring to the different types of solute molecules that contribute to the osmosis, with *N*_*i*_ and *V* being the intracellular number of solute molecules and the cell volume. The permeability coefficient *l*_*m*_ can be regarded as the sum of the permeability coefficients of the lipid bilayer (*l*_*b*_) and the water permeability through aquaporins (*l*_*a*_), *l*_*m*_ = *l*_*b*_ + *l*_*a*_.

A tension pore (tension-induced membrane rupture) occurs after the critical cell volume, corresponding to the critical membrane lateral tension, is reached (Koslov and Markin, 1984). When the tension pore is open, the total volume flow can be written as the sum of the water flows through the cell membrane (*J*_*m*_) and through the tension pore (*J*_*p*_):

(2) $$\frac{\text{d}V}{\text{d}t}=J_{\text{m}}+J_{\text{p}}$$

The volume flow through the tension pore can be described by the equation (Sampson, 1891):

(3) $$J_{\text{p}}=-\frac{R_{\text{p}}^{3}\text{Δ}p}{3\eta }$$

where *R*_*p*_ is the radius of the tension pore and η is the viscosity of the solution.

#### Flows of Solute Molecules

The flows of ions Φ_*m*_*,_*i*_* through the cell membrane can be written as (Hernandez and Chifflet, 2000; Fazelkhah et al., 2018):

(4) $$\Phi_{\text{m},i}=-AP_{i}\Delta c_{i}-AP_{i}\frac{Z_{i}e_{0}}{k_{\text{B}}T}\bar{c_{i}}U+f_{i}A\frac{N}{\Sigma }\left(\alpha -\beta \right)$$

where *P*_*i*_ is the membrane ion permeability of the solute molecules, *Z*_*i*_ is the valence of the charged solutes, *e*_0_ is the elementary charge, $\bar{c_{i}}$ is the mean solute concentration, *U* is the electric potential difference between the interior and the exterior of the cell, *f*_*i*_ is the factor that equals to −3, 2, and 0 for Na^+^, K^+^, and Cl^–^ ions, respectively, *N* is the Na^+^/K^+^-ATPase pump density, Σ is a function of all the rate constants and ligand concentrations, and α and β are functions of the forward and backward rate constants. The first term, which is proportional to the concentration gradient, describes the passive flow of ions. The second term describes the flow of ions induced by the electrostatic potential (Goldman, 1943). The third term describes the flow due to the Na^+^/K^+^-ATPase pumps. The parameter α is proportional to (*N*_*Na*_/V)^3^, *c*_*K*_^2^, and the intracellular ATP concentration, whereas the parameter β is proportional to (*N*_*K*_/V)^2^, *c*_*NA*_^3^, the intracellular concentrations of ADP, and the inorganic phosphate (P_i_) (Fazelkhah et al., 2018). The parameters α and β are also proportional to exp(*e*_0_*U*/2*k*_*B*_*T*) and exp(-*e*_0_*U*/2*k*_*B*_*T*) (Fazelkhah et al., 2018). The constants determining the parameters α and β were adjusted to keep the cell ion concentrations in the growth medium constant. It was also assumed in the model that the numbers of ATP, ADP, and P_i_ remain constant during the time of the simulation.

The flows of solutes Φ_*p*_*,_*i*_* through the tension pore (tension-induced membrane rupture) can be written as (Goldman, 1943; Zemljic Jokhadar et al., 2016; Chabanon et al., 2017):

(5) $$\Phi_{\text{p},i}=\frac{N_{i}}{V}J_{\text{p}}-\pi R_{\text{p}}^{2}D_{i}\frac{\Delta c_{i}}{R_{\text{c}}}-\pi R_{\text{p}}^{2}\frac{Z_{i}e_{0}}{k_{\text{B}}T}\frac{D_{i}}{d}\bar{c_{i}}U$$

where *D*_*i*_ is the diffusion constant of the solute molecules, *R*_*c*_ is the radius of the “cell-vesicle,” and *d* is the membrane thickness. The first term, which is proportional to the volume flow, describes the solute flows due to the convection. The second term, which is proportional to the concentration gradient, describes the solute diffusion. The third term describes the flows of charged solute molecules induced by the electrostatic potential.

The potential difference *U* is determined by the conservation of the electric charge inside the cell, which implies that the sum of all the electric currents of the charged solute molecules must be equal to zero at any time (∑_*i*_*Z*_*i*_(Φ_m,*i*_ + Φ_p,*i*_) = 0).

The total initial extracellular and intracellular concentrations of all the solute molecules were taken to be equal to the physiological concentration (315 mmol/L). Only the most abundant ions (Na^+^, K^+^, and Cl^–^) in the Leibovitz medium with concentrations over 5 mmol/L were considered in the model. The organic osmolytes, e.g., amino acids and inositol, were not considered to enter the cell and were assumed to be on average without any electric charge. The sucrose molecules can enter the cell only by diffusion through the tension pore and leave the cell by diffusion and convection through the tension pore. In the cell, the difference between the physiological concentration and the concentrations of the most abundant intracellular ions (Na^+^, K^+^, and Cl^–^) pertains to the concentration of the molecules in the cell that cannot penetrate the plasma membrane. These molecules were taken to be either too large to penetrate the membrane or they were not taken into account for the membrane penetration because of their small concentrations. These molecules are assumed to be electrically neutral on average and can leave the cell only by the convection through the tension pore. The parameters used are specified in Table 1.

**TABLE 1:** Values of parameters employed in the model.

Parameter	Value
Initial Na^+^ intracellular concentration	11 mmol/L (Fazelkhah et al., 2018)
Initial K^+^ intracellular concentration	145 mmol/L (Fazelkhah et al., 2018)
Initial Cl^–^ intracellular concentration	70 mmol/L (Fazelkhah et al., 2018)
Growth medium Na^+^ concentration	145.4 mmol/L (Gibco, United States; Supplementary Material)
Growth medium K^+^ concentration	5.3 mmol/L (Gibco, United States; Supplementary Material)
Growth medium Cl^–^ concentration	126.1 mmol/L (Gibco, United States; Supplementary Material)
Leibovitz’s medium Na^+^ concentration	140.6 mmol/L (Gibco, United States; Supplementary Material)
Leibovitz’s medium K^+^ concentration	5.7 mmol/L (Gibco, United States; Supplementary Material)
Leibovitz’s medium Cl^–^ concentration	145.9 mmol/L (Gibco, United States; Supplementary Material)
Na^+^ diffusion constant in water (*D*_*Na*_)	1.334 × 10^–9^ m^2^/s (Samson et al., 2003; Velegol et al., 2016)
K^+^ diffusion constant in water (*D*_*K*_)	1.957 × 10^–9^ m^2^/s (Samson et al., 2003; Velegol et al., 2016)
Cl^–^ diffusion constant in water (*D*_*Cl*_)	2.032 × 10^–9^ m^2^/s (Samson et al., 2003; Velegol et al., 2016)
Sucrose diffusion constant in water	0.52 × 10^–9^ m^2^/s (Lide, 2004)
Na^+^ membrane permeability (*P*_*Na*_)	5.0 × 10^–10^ m/s (this study)
K^+^ membrane permeability (*P*_*K*_)	6.2 × 10^–10^ m/s (this study)
Cl^–^ membrane permeability (*P*_*Cl*_)	3.2 × 10^–9^ m/s (Fazelkhah et al., 2018)
Critical membrane tension (σ_*C*_)	1.19 × 10^–2^ N/m (Evans et al., 2003; Hsueh et al., 2007)
Membrane-stretching constant (*k*_*A*_)	0.354 N/m (Tierney et al., 2005)
Line tension of the pore (Γ)	1.7 × 10^–11^ N (Molotkovsky and Akimov, 2009; Portet and Dimova, 2010)
Permeability coefficient of CHO cell membrane (*l*_*m*_)	9 × 10^–14^ m^3^/(Ns) (Farinas et al., 1997)
Viscosity of the solution (η)	0.9 × 10^–3^ Pas
Viscosity of the membrane (η_*m*_)	5 Pas (Hormel et al., 2014)
Temperature (*T*)	310 K
Critical cell vesicle radius [*R*_*C*_ = (3*V*_*C*_/4π)^1/3^]	18 μm
Membrane thickness (*d*)	5 nm

*The corresponding references are listed.*

The values of initial intracellular concentrations for Na^+^, K^+^, and Cl^–^ ions in CHO epithelial cells and the membrane permeability for Cl^–^ ions were taken from the literature (Table 1). In the next step, the Na^+^ and K^+^ membrane permeabilities (*P*_*Na*_ and *P*_*K*_) were adapted to our experimental conditions in the growth medium (Table 1) by taking the net flow of water molecules and ions across the membrane equal to 0 in order to keep the cell volume constant. Namely, the obtained values for *P*_*Na*_ and *P*_*K*_ were tuned in such a way that their mean value (5.6 × 10^–10^ m/s) corresponds to the value used in the literature (Charras et al., 2005).

#### Determination of the Tension Pore Radius

The tension pore occurs when the critical membrane tension (σ_*C*_) is reached, corresponding to the critical hydrostatic pressure (Δ*p*_*C*_ = 2σ_*C*_/*R*_*C*_, with *R*_*C*_ the critical “cell-vesicle” radius). The initial tension pore radius equals to Γ/σ_*C*_, and its temporal evolution can be determined with the differential equation (Brochard-Wyart et al., 2000; Ryham et al., 2011; Aubin Christopher and Ryham Rolf, 2016):

(6) $$\left(d\eta_{\text{m}}+2\pi \eta R_{\text{p}}\right)\frac{\text{d}R_{\text{p}}}{\text{d}t}=\sigma R_{\text{p}}-\Gamma$$

where η_*m*_ and σ are the membrane viscosity and its lateral tension, and Γ is the line tension of the pore. The tension σ depends on the membrane-stretching constant *k*_*A*_ through the equation σ = *k*_*A*_(*A* − *A*_0_)/*A*_0_, where *A* (*A* = 4π*R*_*c*_^2^ − π*R*_*p*_^2^) and *A*_0_ are the expanded and the equilibrium membrane areas.

The values for the constants σ_*C*_, *k*_*A*_, and Γ correspond to the typical values of lipid bilayers (Table 1). The critical cell volume was taken to be 3.4 times the initial one (*V*_*C*_ = 3.4*V*_0_) in agreement with the experimental results. Furthermore, it was assumed that the hydrostatic pressure Δ*p* increases linearly with the cell volume [Δ*p* = (2σ_*C*_/*R*_*C*_)(*V* − *V*_0_)/(*V*_*C*_ − *V*_0_)] until the occurrence of the tension pore. The initial Δ*p* was assumed to be 0.

The role of the cortical actin-based cytoskeleton is not considered in the model although the blebs are well known to reassemble the cortical actin cytoskeleton very quickly (Charras et al., 2006). However, it was demonstrated that “cell-vesicles” possess no actin fibers or any other forms of actin organization, especially in the upper part of the cells that could provide mechanical resistance (Zemljic Jokhadar et al., 2016). Therefore, the contribution of the cytoskeleton contraction to the observed partial ejection of the cytosol cannot be expected to systematically increase the area elasticity modulus of the stretchable bilayer.

### Predictions of the Model

#### Behavior in the Leibovitz-Water Solutions

**FIGURE 9:**
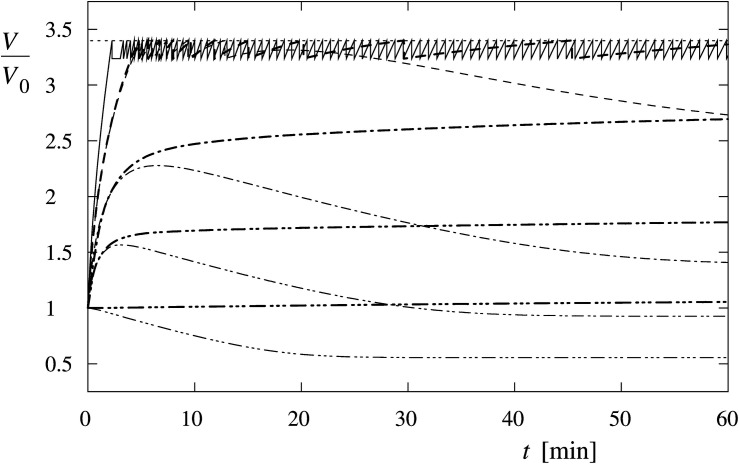
Predicted time behavior of the cell volume at different osmolarities of Leibovitz-water (thick lines) and sucrose-water (thin lines) solutions. It is depicted for osmolarities equal to 189 (dash double dot lines), 126 (dash dot lines), and 63 mosM/L (dashed lines). Iso-osmolar Leibovitz’s medium and iso-osmolar sucrose-water solution (dash triple dot lines) and the distilled water (full line) are also depicted. The dotted line indicates the critical cell volume. The cell volume is normalized to its initial volume (*V*/*V*_0_).

**FIGURE 10:**
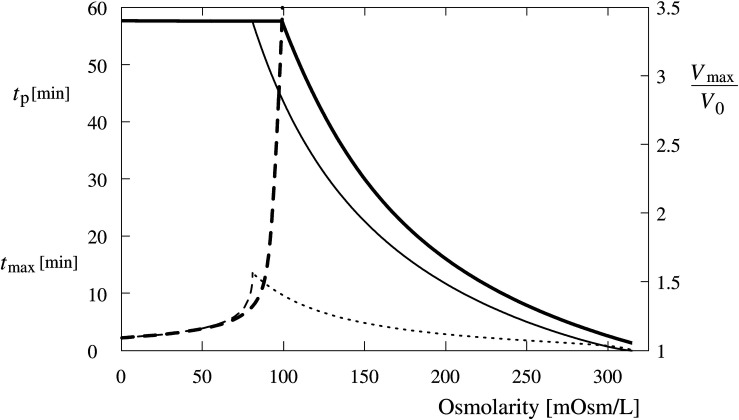
Predicted occurrence time (*t*_*p*_) of the first tension pore (dashed lines) and maximum cell volume (*V*_*max*_/*V*_0_) (full lines) as a function of the osmolarity of Leibovitz-water (thick lines) and sucrose-water (thin lines) solutions. In addition, the time of the maximum volume of the cell (*t*_*max*_) in the sucrose-water solution is shown by the thin dotted line. The maximum cell volume is plotted relative to its initial value.

The theoretical results are obtained from numerical solutions of the system of equations [Eqs. (1) to (6)]. The water inflow, driven essentially by the osmotic pressure, induces only a small increase in the cell volume in Leibovitz-water solutions with low osmolarity gradients (Figure 9). Consequently, asymptotic increases of the cell’s volume to its maximum value at the end of the 1-h period are predicted for higher osmolarities (189 and 126 mosM/L), which can be seen in Figure 9 by the thick dash double dot (189 mosM/L) and dash dot (126 mosM/L) lines. In general, steeper increases in the cell volume are predicted at decreasing osmolarities. The maximum values of the cell volume within the simulation period are shown in Figure 10.

In contrary, as seen in Figure 9 for the osmolarity of the Leibovitz-water solution equal to 63 mosM/L (thick dashed line) and for the distilled water (full line), the cell’s volume increases up to the critical cell volume are predicted. Consequently, a tension pore (tension-induced membrane rupture) occurs at the critical volume, and the cell volume diminishes. After the closure of the tension pore, the cell’s volume starts to increase again and can attain its critical volume again. In this way, the process of the tension pore occurrence can be repeated.

The critical cell volume is reached in cells exposed to the Leibovitz-water solutions with osmolarities lower than 99 mosM/L (Figure 10, full thick line). The predicted occurrence time of the tension pore increases with the increasing osmolarity of the Leibovitz-water solution. For example, the tension pore occurrence times equal to 2 min are predicted for the distilled water and 5 and 34 min for the Leibovitz-water solutions with osmolarities equal to 63 and 95 mosM/L, as seen by the dashed thick line in Figure 10. The increase of the tension pore occurrence time becomes significantly steeper at osmolarities higher than around 80 mosM/L. It has also to be mentioned that the tension pore opening times, predicted by the model for the first tension pore, decrease with the increasing osmolality of the Leibovitz-water solutions (not shown). It has to be noted that the predicted occurrence times also depend on the water permeability of the membrane (see Supplementary Material).

The duration of the tension pores is also dependent on the line tension of the pore. The simulations show that an increased line tension increases the number of consecutive tension pores and lowers their duration. For example, an increase of Γ value from 1.7 × 10^–11^ to 3.0 × 10^–11^ N raises the number of tension pores from 82 to 86, whereas its decrease to the value of 1.0 × 10^–11^ N decreases the number of tension pores to 50 in the distilled water.

It has also to be noted that the results predicted for the iso-osmolar undiluted Leibovitz’s medium show a minor relative increase of cell’s volume of 5% (Figures 9, 10).

#### Behavior in the Sucrose-Water Solutions

The theoretical results obtained for the sucrose-water solutions predict a significantly different behavior compared with the Leibovitz-water solutions. A significant decrease of the cell’s volume to a 56% of its initial volume is encountered already in the iso-osmolar sucrose-water solution, which differs from the one predicted for the iso-osmolar Leibovitz’s solution, as seen by the dash triple dot lines in Figure 9.

Accordingly, the results of the simulations in the sucrose-water solutions with the osmolarities corresponding to Leibovitz-water solutions showed significant differences in the predicted volume increase, its maximum value, as well as in the predicted occurrence time of the tension pores and their temporal behavior (Figures 9, 10). The increases of the cell’s volume in the sucrose-water solutions with higher osmolarities (189 and 126 mosM/L), which can be seen in Figure 9 by the thin dash double dot (189 mosM/L) and dash dot (126 mosM/L) lines, experience comparable cell’s volume increases within the first minutes of the simulation period. However, significant decreases in the cell’s volume are predicted afterward, and accordingly, the maximum values of the cell’s volume do not occur at the end of the 1-h period. The maximum values of the cell’s volume and their occurrence times are shown in Figure 10.

The cell’s volume increases up to the critical volume and tension pore occurrences are predicted for osmolarities of the sucrose-water solutions lower than 81 mosM/L (Figures 9, 10). The cell’s volume can significantly diminish after the occurrence of one or several tension pores as demonstrated in Figure 9 by the thin dashed line. The tension pores in the sucrose-water solutions are predicted to occur only at times shorter than 14 min, depending on the osmolarity of the solutions.

#### Reliability of the Model

The Goldman approach was used in the model [Eqs. (4) and (5)] to describe the flow of ions induced by the electrostatic potential (Goldman, 1943). The simulations show that if we use the Hodgkin Katz approach to model the flow of ions induced by the electrostatic potential (Hodgkin and Katz, 1949), the predicted differences in cell’s volume increase and tension pore occurrence time compared with the ones using Goldman approach are non-significant. For instance, when the Hodgkin Katz approach is used, only less than 0.2% higher relative increase of the cell’s volume is predicted in the Leibovitz’s medium and in the 185 mosM/L Leibovitz-water solution. Correspondingly, the first tension pore is predicted to occur at less than 0.02% shorter times in 63 and 32 mosM/L Leibovitz-water solutions when the Hodgkin Katz approach is used instead of the Goldman approach.

It was also assumed that the hydrostatic pressure Δ*p* increases linearly with the cell volume (*V*) from its initial value (*V*_0_) to its critical value (*V*_*C*_). Even though the pressure is expected to remain more or less unchanged during the initial swelling since some portion of the cell membrane is not tightly attached to the substrate or to the cytoskeleton, it can be shown that the alterations of the assumed pressure behavior do not significantly affect the predictions of the model. The simulations show that either a prompt increase of the hydrostatic pressure to its critical value (Δ*p* = 2σ_*C*_/*R*_*C*_) appears at the beginning or a slow one with an almost negligible pressure value (Δ*p* = 0) till the pore formation is assumed, only 1% difference in the cell’s volume variation is predicted in a 158 mosM/L Leibovitz-water solution. This demonstrates a robustness of the model concerning the dependency of Δ*p* on the cell volume. As a consequence, it is not possible to assess the intracellular pressure changes using this model.

For the numerical simulations, the value of the area elasticity modulus *k*_*A*_, typical for lipid bilayers, was used. The results show that the effect of *k*_*A*_ on relative volume changes is not pronounced, which makes the model stable for a larger range of *k*_*A*_ values in the literature (Pietuch et al., 2013). For example, in 158 mosM/L Leibovitz-water solution at 10 times smaller *k*_*A*_ value, only 0.5% lower value of the maximum volume is predicted, and at 10 times higher *k*_*A*_ value, only 0.1% higher value of this volume is predicted, respectively.

The program can be found on the GitHub^1^.

## Discussion

The goal of this study was to examine the cell’s volume changes and the occurrence of tension-induced membrane ruptures in hypotonic media. The experimental results show that the characteristic cell behavior depends strongly on the osmolarity of the medium, and that it differs considerably between the electrolyte and sugar solutions. A theoretical model presented can explain the essential characteristics of the observed phenomena.

### Experimental and Theoretical Results

An inflow of water into the cell is induced as a result of the osmotic pressure difference in a hypotonic medium. Because of the limited capacity of the cell’s active self-preservation mechanisms, a volume increase is expected. Concomitantly, the changes in the cell’s shape and footprint, the formation and merging of blebs, and the occurrence of a single bleb (i.e., “cell-vesicle”) are experienced (Figures 2, 3). These phenomena are expressed in a more spherical cell shape, which optimizes the cell volume at a given membrane area (Groulx et al., 2006).

The theory predicts comparable short-term increases of the observed cell’s volume in hypotonic electrolyte and sugar solutions at higher osmolarities (Figures 4, 9). They also predict the observed long-time volume differences between the hypotonic electrolyte and sugar solutions. The higher efficiency of the RVD in a modest osmotically induced water inflow is namely reflected in “type D” cell’s behavior pattern in sucrose-water solutions and in an asymptotically increasing “type A” volume behavior pattern in electrolyte Leibovitz-water solutions (Figure 6). In accordance, significant differences in the occurrence times in electrolyte and sugar solutions can be foreseen by the model (Figure 10). A good correlation of the predicted volume decrease is also obtained in the iso-osmolar sucrose solution where an approximately 50% cell’s volume decrease was observed in a period of 1 h (Figures 4B, 9).

In addition, the model defines the role of tension-induced membrane ruptures at lower osmolarities and explains the observed differences in the osmolarity range and the occurrence times in the electrolyte and sugar solutions (cf. Figures 4, 10). Namely, the self-preservation mechanisms are not sufficient to compensate the intensive osmotically induced water inflow at lower osmolarities. In this case, the cells’ volumes grow the most intensively (Figures 4, 5), and “cell-vesicles” are formed (Figure 2, fifth row). The “cell-vesicle” is regarded as the ultimate stage where all the hidden membrane reserves are exhausted.

When the cell’s volume equals to approximately 3.4 times the initial cell volume (Figure 4), the membrane’s lateral tension reaches its critical value, and a membrane rupture occurs as seen in Supplementary Videos 1, 2 (Brochard-Wyart et al., 2000; Kristanc et al., 2014; Zemljic Jokhadar et al., 2016; Chabanon et al., 2017). Short or longer lasting membrane ruptures are predicted to repeat until the osmolarities inside and outside the cell are essentially equalized (Figure 9). The longer lasting membrane rupture is characterized by a quick increase and partial decrease of the pore radius that stabilizes at a significantly lower value for a longer period. It is predicted at the lowest osmolarities where the highest water inflows cannot be compensated by a short membrane opening. With the occurrence of a non-selective leakage of the cell contents during the membrane rupture (Figure 3 and Supplementary Video 1), the observed characteristic “type B” and “type C” time-behavior patterns of the cell volume can be explained (Figure 6). After the closure of a membrane rupture, the cell’s volume can start to increase again, and the process of the membrane rupture occurrence can be repeated that explains the “type B” time-behavior pattern (Figures 6, 9). The “type C” time-behavior pattern, which is characterized by a significant decrease of the cell’s critical volume, could be correlated to the “cell-vesicles” that resealed completely or partially after longer lasting membrane rupture (Supplementary Video 1 and Figures 6, 9). The occurrence of membrane ruptures, which were observed either during the whole measuring time in the electrolyte Leibovitz-water solutions or within a limited time after the exposure to the sucrose-water solutions, is also predicted by the model (Figures 7, 10). The latter could also explain the loss of the fluorescent signal observed during the whole measuring time at higher osmolarity values in the Leibovitz-water solutions (Figures 7, 10).

The data found in the literature, in particular on CHO cells, are scarce and partially divergent. A fast increase of the cell’s volume was observed in CHO cells when exposed to 6 mosM/L electrolyte solution (Groulx et al., 2006). Critical cell volume increases equal to approximately 10 times the initial cell volume were determined, and the occurrence times of membrane ruptures were found to be around 5 min after the cell exposure to the hypotonic medium (Groulx et al., 2006). Furthermore, an average maximum cell volume increase equal to 1.3 times the initial cell volume was obtained 2 min after the exposure of CHO cells to 100 mosM/L sucrose solution. The temporal behavior of the cell’s volume is reflected in the “type D” behavior pattern (Usaj et al., 2009). Hence, the occurrence times of membrane ruptures and of maximum volumes are in accordance with our observations (Figure 4); however, the maximal value is either significantly higher (Groulx et al., 2006) or significantly lower (Usaj et al., 2009) than our results. The large differences in the results will be addressed in the Biological Aspects section. It is also to be noted that the Boyle van’t Hoff plot cannot be used to estimate the osmolality range at which membrane rupture occurs in our case since it is based on the concept of the constant amount of the intracellular impermeable solute (Katkov, 2011).

### Reasons for the Differences in the Hypotonic Electrolyte and Sugar Solutions

The theoretical model incorporates (i) the effects induced by the osmosis and the hydrostatic pressure that drive water molecules into the cell, (ii) the transmembrane movements of Na^+^, K^+^, and Cl^–^ due to their intracellular and extracellular concentration differences and due to the active Na^+^/K^+^-ATPase pumps, (iii) and the occurrence of tension-induced membrane ruptures in the membrane with the corresponding flows (Koslov and Markin, 1984). The observed differences in the behavior of cells in the Leibovitz-water solutions (ion-containing medium) and in the sucrose-water solutions (sugar-containing solution) can be explained based on a high membrane permeability to water, the lower membrane permeabilities to the K^+^, Cl^–^, and Na^+^ ions, and the poor membrane permeability to the sucrose molecules (Reuss et al., 2004; Kiesel et al., 2006; Hoffmann, 2011). The time changes of the cell’s volume can be divided in alignment with Reuss et al. (2004) into two phases: (i) the first phase is characterized by the cell’s volume increase caused by the inflow of water molecules driven by the osmotic difference between the cell and the medium applied. In this phase, only negligible influences on the osmotic difference and, accordingly, on the cell’s volume are expected to be induced by the repositioning of the solute molecules across the cell membrane because of their low membrane permeability. (ii) The second phase is characterized by an influence of the active outflow of Na^+^ and the active inflow of K^+^ ions as well as the passive outflows of K^+^ and Cl^–^ ions and the passive inflow of Na^+^ ions.

In the Leibovitz-water solutions, the active and passive K^+^ and Na^+^ exchange mechanisms experience partially compensating effects on the cell osmolarity. The outflow of Cl^–^ ions is also not pronounced because of the restriction of the cell’s electric neutrality. Consequently, the cell osmolarity does not change significantly, and the cell continues to swell. In contrast, in the sucrose-water solutions, the active flow of ions is disabled, and no passive inflow of sucrose molecules occurs because of their poor membrane permeability. As a result, only outflows of ions occur, and consequently, the cell osmolarity decreases accompanied by an immediate outflow of the water molecules. Hence, the volume decrease will be induced, and a more effective RVD is experienced in the sucrose-water solutions.

The cell’s volume is expected to increase equally in Leibovitz-water and sucrose-water solutions during the first phase, and its rate of increase will be dependent solely on the osmolarity difference between the interior and exterior of the cell. In accordance, a tension-induced membrane rupture will occur in both media at the lowest osmolarities, where the cell volume will increase to the critical volume already during the first phase (Figure 10). Longer occurrence times of membrane ruptures can be expected only in the Leibovitz-water solutions due to an asymptotic increase in the cell volume (Figures 4, 10).

In control experiments undertaken with the Leibovitz’s medium, only minimal changes of the cell’s volume result from a minor water inflow and active and/or passive flows of ions because of the iso-osmolarity and comparable composition to the growth medium (Figure 9). In contrast, cell shrinkage is expected in the iso-osmolar sucrose-water solution due to the passive outflow of K^+^ and Cl^–^ ions without the inflow Na^+^ (Figure 9).

The measurements were also undertaken on the cells, which were pretreated by ouabain in order to eliminate the ion transport through Na^+^/K^+^-ATPase pumps. The cells showed an increase of their volume already after the ouabain treatment (Strange, 1989; Fels et al., 2009). When they were exposed to the Leibovitz’s solution diluted by 60% with distilled water or to the sucrose solution with a comparable osmolarity, the differences in the characteristic time courses were observed. More specifically, lower cell volumes were determined at longer times for the sucrose solution than for the diluted Leibovitz’s solution.

### Biological Aspects

The observed flattening of the membrane and the bleb formation in cells exposed to hypo-osmotic stress are in accordance with the findings in the literature (Ebner et al., 2005; Charras et al., 2008; Babiychuk et al., 2011; Mayor, 2011; Ryham et al., 2011). Larger blebs are formed at volume increases higher than approximately 70%, whereas the “cell-vesicles” are formed at the volume increases higher than approximately 150% (Figures 2, 5). The membrane ruptures that are formed at the critical volume increase (approximately 240%) are essentially the only way by which the cellular macromolecules can leave the cell. The results of the viability presented in Figure 8 show that the seemingly preserved membrane integrity cannot be simply correlated to the cell’s structural and functional integrity as a whole.

The extremely low osmolarities used in the experiments were applied, in spite of the fact that they lead to 100% cell mortality, with the intention to model the cell behavior in a wider range of osmotic gradients. In this way, we could get additional insights into the relation between the cell volume increase and the occurrence of tension-induced membrane ruptures and, concomitantly, the corresponding cell volume maximum. Similar bleb formations, cell detachment, volume increase, and the occurrence of membrane ruptures were namely observed and predicted in CHO cells after the application of the pore-forming antibiotic nystatin (Zemljic Jokhadar et al., 2016).

The scattering of the maximum values measured in individual cells with standard deviations around 35% indicates a large intrinsic variability of the cells (Figure 5 and Table 2). Even a wider range of the cell’s volume increase is expected concerning different cell types where in addition to the differences in experimental parameters used, the differences in the membrane reservoir availability, in the cytoskeleton organization, and in the cell volume regulation mechanisms may take place (Lang et al., 1998; Pasantes-Morales and Tuz, 2006). Nevertheless, since the theoretical model involves the essential contributions to the transmembrane flows through the cell membrane, it can be regarded as a basic quantitative tool for the description of the RVD in the cells.

**TABLE 2:** Standard deviations of the relative volume changes of the cells presented in Figure 4.

*t* (min)	Osmolarities of Leibovitz-water solutions				Osmolarities of sucrose solutions				Distilled water
	315 mosM/L	189 mosM/L	126 mosM/L	63 mosM/L	315 mosM/L	189 mosM/L	126 mosM/L	63 mosM/L	
2		0.30	0.20	0.64		0.41	0.30	0.81	1.07
5	0.10	0.27	0.24	0.48	0.09	0.42	0.23	0.65	1.17
10	0.13	0.30	0.36	0.49	0.08	0.44	0.29	0.54	1.41
60	0.14	0.71	1.22	0.86	0.28	0.43	0.79	0.71	1.44

## Conclusion

Our results clearly show that the *in vitro* results of the volume response to the osmotic shock gained in experiments using different sugar and electrolyte solutions are difficult to be interrelated. The most appropriate time for the comparison is within the first 3 min after the hypotonic shock when the flow of the water is still not significantly influenced by the type of the medium. The observed cell behavior in hypotonic electrolyte and sucrose solutions could be explained by a theoretical model based on the passive transmembrane movement of ions and on the action of Na^+^/K^+^-ATPase pumps. To our knowledge, it is the only quantitative model simulating the cell behavior in the hypotonic conditions that includes the formation of tension-induced membrane ruptures.

## Data Availability Statement

The original contributions generated for this study are included in the article/Supplementary Material, further inquiries can be directed to the corresponding author/s.

## Author Contributions

GG, BB, ŠZJ, and LK: conceptualization, validation, and writing. BB and ŠZJ: analysis and visualization. ŠZJ: realization of experiments. All authors contributed to the article and approved the submitted version.

## Conflict of Interest

The authors declare that the research was conducted in the absence of any commercial or financial relationships that could be construed as a potential conflict of interest.

## Footnotes

1 https://github.com/bbozic-ul/program

## Supplementary Material

Supplementary Video 1 Non-selective leakage of the cell content, observed with bright-field microscopy.

Supplementary Video 2 Fluorescent dye leakage through the tension pore.

## References

[B1] Aubin Christopher A. Ryham Rolf J. (2016). Stokes flow for a shrinking pore. *J. Fluid Mech.* 788 228–245. 10.1017/jfm.2015.699

[B2] Babiychuk E. B. Monastyrskaya K. Potez S. Draeger A. (2011). Blebbing confers resistance against cell lysis. *Cell Death Differ.* 18 80–89. 10.1038/cdd.2010.81 20596076 PMC3131879

[B3] Bloom M. Evans E. Mouritsen O. G. (1991). Physical properties of the fluid lipid-bilayer component of cell membranes: a perspective. *Q. Rev. Biophys.* 24 293–397. 10.1017/s0033583500003735 1749824

[B4] Brochard-Wyart F. de Gennes P. G. Sandre O. (2000). Transient pores in stretched vesicles: role of leak-out. *Phys. A Stat. Mech. Appl.* 278 32–51. 10.1016/s0378-4371(99)00559-2

[B5] Burg M. B. Garcia-Perez A. (1992). How tonicity regulates gene expression. *J. Am. Soc. Nephrol.* 3 121–127. 1391714 10.1681/ASN.V32121

[B6] Caruccio L. Bae S. Liu A. Y. Chen K. Y. (1997). The heat-shock transcription factor HSF1 is rapidly activated by either hyper- or hypo-osmotic stress in mammalian cells. *Biochem. J.* 327(Pt 2), 341–347. 10.1042/bj3270341 9359399 PMC1218799

[B7] Chabanon M. Ho J. C. S. Liedberg B. Parikh A. N. Rangamani P. (2017). Pulsatile lipid vesicles under osmotic stress. *Biophys. J.* 112 1682–1691. 10.1016/j.bpj.2017.03.018 28445759 PMC5406380

[B8] Charras G. T. Coughlin M. Mitchison T. J. Mahadevan L. (2008). Life and times of a cellular bleb. *Biophys. J.* 94 1836–1853. 10.1529/biophysj.107.113605 17921219 PMC2242777

[B9] Charras G. T. Hu C. K. Coughlin M. Mitchison T. J. (2006). Reassembly of contractile actin cortex in cell blebs. *J. Cell. Biol.* 175 477–490. 10.1083/jcb.200602085 17088428 PMC2064524

[B10] Charras G. T. Yarrow J. C. Horton M. A. Mahadevan L. Mitchison T. J. (2005). Non-equilibration of hydrostatic pressure in blebbing cells. *Nature* 435 365–369. 10.1038/nature03550 15902261 PMC1564437

[B11] Culliford S. J. Borg J. J. O’Brien M. J. Kozlowski R. Z. (2004). Differential effects of pyrethroids on volume-sensitive anion and organic osmolyte pathways. *Clin. Exp. Pharmacol. Physiol.* 31 134–144. 10.1111/j.1440-1681.2004.03965.x 15008955

[B12] de Los Heros P. Pacheco-Alvarez D. Gamba G. (2018). Role of WNK kinases in the modulation of cell volume. *Curr. Top. Membr.* 81 207–235. 10.1016/bs.ctm.2018.08.002 30243433

[B13] Duan D. Winter C. Cowley S. Hume J. R. Horowitz B. (1997). Molecular identification of a volume-regulated chloride channel. *Nature* 390 417–421. 10.1038/37151 9389484

[B14] Ebner H. L. Cordas A. Pafundo D. E. Schwarzbaum P. J. Pelster B. Krumschnabel G. (2005). Importance of cytoskeletal elements in volume regulatory responses of trout hepatocytes. *Am. J. Physiol. Regul. Integr. Comp. Physiol.* 289 R877–R890. 15905223 10.1152/ajpregu.00170.2005

[B15] Echarri A. Del Pozo M. A. (2015). Caveolae - mechanosensitive membrane invaginations linked to actin filaments. *J. Cell. Sci.* 128 2747–2758. 10.1242/jcs.153940 26159735

[B16] Echarri A. Pavón D. M. Sánchez S. García-García M. Calvo E. Huerta-López C. (2019). An Abl-FBP17 mechanosensing system couples local plasma membrane curvature and stress fiber remodeling during mechanoadaptation. *Nat. Commun.* 10:5828. 10.1038/s41467-019-13782-2 PMC6925243 31862885

[B17] Evans E. Heinrich V. Ludwig F. Rawicz W. (2003). Dynamic tension spectroscopy and strength of biomembranes. *Biophys. J.* 85 2342–2350. 10.1016/s0006-3495(03)74658-x 14507698 PMC1303459

[B18] Farinas J. Kneen M. Moore M. Verkman A. S. (1997). Plasma membrane water permeability of cultured cells and epithelia measured by light microscopy with spatial filtering. *J. Gen. Physiol.* 110 283–296. 10.1085/jgp.110.3.283 9276754 PMC2229369

[B19] Fazelkhah A. Braasch K. Afshar S. Salimi E. Butler M. Bridges G. (2018). Quantitative model for ion transport and cytoplasm conductivity of chinese hamster ovary cells. *Sci. Rep.* 8:17818. 10.1038/s41598-018-36127-3 PMC6292909 30546044

[B20] Fels J. Orlov S. N. Grygorczyk R. (2009). The hydrogel nature of mammalian cytoplasm contributes to osmosensing and extracellular pH sensing. *Biophys. J.* 96 4276–4285. 10.1016/j.bpj.2009.02.038 19450498 PMC2712211

[B21] Goldman D. E. (1943). Potential, impendance, and rectification in mmembranes. *J. Gen. Physiol.* 27 37–60. 10.2307/1296184 19873371 PMC2142582

[B22] Groulx N. Boudreault F. Orlov S. N. Grygorczyk R. (2006). Membrane reserves and hypotonic cell swelling. *J. Membr. Biol.* 214 43–56. 10.1007/s00232-006-0080-8 17598067

[B23] Hall J. A. Kirk J. Potts J. R. Rae C. Kirk K. (1996). Anion channel blockers inhibit swelling-activated anion, cation, and nonelectrolyte transport in HeLa cells. *Am. J. Physiol.* 271(2 Pt 1), C579–C588. 8769998 10.1152/ajpcell.1996.271.2.C579

[B24] Hamill O. P. Martinac B. (2001). Molecular basis of mechanotransduction in living cells. *Physiol. Rev.* 81 685–740. 10.1152/physrev.2001.81.2.685 11274342

[B25] Hernandez J. A. Chifflet S. (2000). Electrogenic properties of the sodium pump in a dynamic model of membrane transport. *J. Membr. Biol.* 176 41–52. 10.1007/s002320001074 10882427

[B26] Hodgkin A. L. Katz B. (1949). The effect of sodium ions on the electrical activity of giant axon of the squid. *J. Physiol.* 108 37–77. 10.1113/jphysiol.1949.sp004310 18128147 PMC1392331

[B27] Hoffmann E. K. (2011). Ion channels involved in cell volume regulation: effects on migration, proliferation, and programmed cell death in non adherent EAT cells and adherent ELA cells. *Cell Physiol. Biochem.* 28 1061–1078. 10.1159/000335843 22178996

[B28] Hohmann S. (2015). An integrated view on a eukaryotic osmoregulation system. *Curr. Genet.* 61 373–382. 10.1007/s00294-015-0475-0 25663258

[B29] Hormel T. T. Kurihara S. Q. Brennan M. K. Wozniak M. C. Parthasarathy R. (2014). Measuring lipid membrane viscosity using rotational and translational probe diffusion. *Phys. Rev. Lett.* 112:188101. 10.1103/PhysRevLett.112.188101 24856725

[B30] Hsueh Y. W. Chen M. T. Patty P. J. Code C. Cheng J. Frisken B. J. (2007). Ergosterol in POPC membranes: physical properties and comparison with structurally similar sterols. *Biophys. J.* 92 1606–1615. 10.1529/biophysj.106.097345 17142279 PMC1796827

[B31] Huang H. W. Chen F. Y. Lee M. T. (2004). Molecular mechanism of Peptide-induced pores in membranes. *Phys. Rev. Lett.* 92:198304. 10.1103/PhysRevLett.92.198304 15169456

[B32] Jackson P. S. Strange K. (1993). Volume-sensitive anion channels mediate swelling-activated inositol and taurine efflux. *Am. J. Physiol.* 265(6 Pt 1), C1489–C1500. 8279513 10.1152/ajpcell.1993.265.6.C1489

[B33] Jennings M. L. Schulz R. K. (1990). Swelling-activated KCl cotransport in rabbit red cells: flux is determined mainly by cell volume rather than shape. *Am. J. Physiol.* 259(6 Pt 1), C960–C967. 2260643 10.1152/ajpcell.1990.259.6.C960

[B34] Katkov I. I. (2011). On proper linearization, construction and analysis of the Boyle-van’t Hoff plots and correct calculation of the osmotically inactive volume. *Cryobiology* 62 232–241. 10.1016/j.cryobiol.2011.02.006 21376029

[B35] Kiesel M. Reuss R. Endter J. Zimmermann D. Zimmermann H. Shirakashi R. (2006). Swelling-activated pathways in human T-lymphocytes studied by cell volumetry and electrorotation. *Biophys. J.* 90 4720–4729. 16565059 10.1529/biophysj.105.078725 PMC1471856

[B36] Kirkegaard S. S. Strøm P. D. Gammeltoft S. Hansen A. J. Hoffmann E. K. (2016). The volume activated potassium channel KCNK5 is up-regulated in activated human T cells, but volume regulation is impaired. *Cell Physiol. Biochem.* 38 883–892. 10.1159/000443042 26909737

[B37] Koslov M. M. Markin V. S. (1984). A theory of osmotic lysis of lipid vesicles. *J. Theor. Biol.* 109 17–39. 10.1016/s0022-5193(84)80108-3 6471867

[B38] Kristanc L. Bozic B. Gomiscek G. (2014). The role of sterols in the lipid vesicle response induced by the pore-forming agent nystatin. *Biochim. Biophys. Acta* 1838 2635–2645. 10.1016/j.bbamem.2014.05.019 24863056

[B39] Lang F. Busch G. L. Volkl H. (1998). The diversity of volume regulatory mechanisms. *Cell Physiol. Biochem.* 8 1–45. 10.1159/000096284 9547017

[B40] Lide D. R. (2004). *CRC Handbook of Chemistry and Physics.* Boca Raton, FL: CRC press.

[B41] Liviu I. G. Dumitru P. (2019). A mathematical investigation on the active substance pulsatory release from a solution-charged liposome. *Biosystems* 179 48–54. 10.1016/j.biosystems.2019.03.001 30851346

[B42] Mally M. Majhenc J. Svetina S. Zeks B. (2007). The response of giant phospholipid vesicles to pore-forming peptide melittin. *Biochim. Biophys. Acta* 1768 1179–1189. 10.1016/j.bbamem.2007.02.015 17383608

[B43] Mayor S. (2011). Need tension relief fast? Try Caveolae. *Cell* 144 323–324. 10.1016/j.cell.2011.01.018 21295694

[B44] McManus M. L. Churchwell K. B. Strange K. (1995). Regulation of cell volume in health and disease. *N. Engl. J. Med.* 333 1260–1266. 10.1056/nejm199511093331906 7566004

[B45] Molotkovsky R. J. Akimov S. A. (2009). Calculation of line tension in various models of lipid bilayer pore edge. *Biochemistry Suppl. Ser. A Membr. Cell Biol.* 3 223–230. 10.1134/s1990747809020160

[B46] Nichol J. A. Hutter O. F. (1996). Tensile strength and dilatational elasticity of giant sarcolemmal vesicles shed from rabbit muscle. *J. Physiol.* 493(Pt 1), 187–198. 10.1113/jphysiol.1996.sp021374 8735704 PMC1158960

[B47] Okada Y. Maeno E. Shimizu T. Dezaki K. Wang J. Morishima S. (2001). Receptor-mediated control of regulatory volume decrease (RVD) and apoptotic volume decrease (AVD). *J. Physiol.* 532(Pt 1), 3–16. 10.1111/j.1469-7793.2001.0003g.x 11283221 PMC2278524

[B48] Orlov S. N. Shiyan A. Boudreault F. Ponomarchuk O. Grygorczyk R. (2018). Search for upstream cell volume sensors: the role of plasma membrane and cytoplasmic hydrogel. *Curr. Top. Membr.* 81 53–82. 10.1016/bs.ctm.2018.07.001 30243440

[B49] Pasantes-Morales H. Tuz K. (2006). Volume changes in neurons: hyperexcitability and neuronal death. *Contrib. Nephrol.* 152 221–240. 10.1159/000096326 17065815

[B50] Pietuch A. Bruckner B. R. Janshoff A. (2013). Membrane tension homeostasis of epithelial cells through surface area regulation in response to osmotic stress. *Biochim. Biophys. Acta* 1833 712–722. 23178740 10.1016/j.bbamcr.2012.11.006

[B51] Portet T. Dimova R. (2010). A new method for measuring edge tensions and stability of lipid bilayers: effect of membrane composition. *Biophys. J.* 99 3264–3273. 10.1016/j.bpj.2010.09.032 21081074 PMC2980741

[B52] Qiu Z. Dubin A. E. Mathur J. Tu B. Reddy K. Miraglia L. J. (2014). SWELL1, a plasma membrane protein, is an essential component of volume-regulated anion channel. *Cell* 157 447–458. 10.1016/j.cell.2014.03.024 24725410 PMC4023864

[B53] Reuss R. Ludwig J. Shirakashi R. Ehrhart F. Zimmermann H. Schneider S. (2004). Intracellular delivery of carbohydrates into mammalian cells through swelling-activated pathways. *J. Membr. Biol.* 200 67–81. 10.1007/s00232-004-0694-7 15520905

[B54] Ryham R. Berezovik I. Cohen F. S. (2011). Aqueous viscosity is the primary source of friction in lipidic pore dynamics. *Biophys. J.* 101 2929–2938. 10.1016/j.bpj.2011.11.009 22208191 PMC3244058

[B55] Sampson R. A. (1891). On stokes’s current function. *Philos. Trans. R. Soc. Lond. A* 182 449–518.

[B56] Samson E. Marchand J. Snyder K. A. (2003). Calculation of ionic diffusion coefficients on the basis of migration test results. *Mater. Struct.* 36 156–165. 10.1617/14002

[B57] Sarkadi B. Parker J. C. (1991). Activation of ion transport pathways by changes in cell volume. *Biochim. Biophys. Acta* 1071 407–427. 10.1016/0304-4157(91)90005-h 1721542

[B58] Strange K. (1989). Ouabain-induced cell swelling in rabbit cortical collecting tubule: NaCl transport by principal cells. *J. Membr. Biol.* 107 249–261. 10.1007/bf01871940 2716047

[B59] Strange K. (2004). Cellular volume homeostasis. *Adv. Physiol. Educ.* 28 155–159. 10.1152/advan.00034.2004 15545344

[B60] Strange K. Jackson P. S. (1995). Swelling-activated organic osmolyte efflux: a new role for anion channels. *Kidney Int.* 48 994–1003. 10.1038/ki.1995.381 8569109

[B61] Stutzin A. Torres R. Oporto M. Pacheco P. Eguiguren A. L. Cid L. P. (1999). Separate taurine and chloride efflux pathways activated during regulatory volume decrease. *Am. J. Physiol.* 277 C392–C402. 10484326 10.1152/ajpcell.1999.277.3.C392

[B62] Tierney K. J. Block D. E. Longo M. L. (2005). Elasticity and phase behavior of DPPC membrane modulated by cholesterol, ergosterol, and ethanol. *Biophys. J.* 89 2481–2493. 10.1529/biophysj.104.057943 16055540 PMC1366747

[B63] Usaj M. Trontelj K. Hudej R. Kanduser M. Miklavcic D. (2009). Cell size dynamics and viability of cells exposed to hypotonic treatment and electroporation for electrofusion optimization. *Radiol. Oncol.* 43 108–119.

[B64] Velegol D. Garg A. Guha R. Kar A. Kumar M. (2016). Origins of concentration gradients for diffusiophoresis. *Soft. Matter.* 12 4686–4703. 10.1039/c6sm00052e 27174044

[B65] Voss F. K. Ullrich F. Münch J. Lazarow K. Lutter D. Mah N. (2014). Identification of LRRC8 heteromers as an essential component of the volume-regulated anion channel VRAC. *Science* 344 634–638. 10.1126/science.1252826 24790029

[B66] Zemljic Jokhadar S. Bozic B. Kristanc L. Gomiscek G. (2016). Osmotic effects induced by pore-forming agent nystatin: from lipid vesicles to the cell. *PLoS One* 11:e0165098. 10.1371/journal.pone.0165098 27788169 PMC5082891

