# Supplementary material for: Cell Volume Changes and Membrane Ruptures Induced by Hypotonic Electrolyte and Sugar Solutions
Source: Front Physiol. 2020 Dec 7;11:582781. doi: 10.3389/fphys.2020.582781 (PMC7750460; doi:10.3389/fphys.2020.582781)
Supplement: Supplementary file 3 [file Table_1.DOCX]

Supplementary

Supplementary 1. Tables of Leibovitz's L-15 and Advanced MEM solutions.

Table S1. Leibovitz's L-15.

| **Components** | **Molecular Weight (g/mol)** | **Concentration (mg/L)** | **Concentration(mM)** |
| --- | --- | --- | --- |
| **Amino Acids** | | | |
| Glycine | 75.0 | 200.0 | 2.6666667 |
| L-Alanine | 89.0 | 225.0 | 2.52809 |
| L-Arginine | 174.0 | 500.0 | 2.8735633 |
| L-Asparagine | 132.0 | 250.0 | 1.8939394 |
| L-Cysteine | 121.0 | 120.0 | 0.9917355 |
| L-Glutamine | 146.0 | 300.0 | 2.0547945 |
| L-Histidine | 155.0 | 250.0 | 1.6129032 |
| L-Isoleucine | 131.0 | 250.0 | 1.908397 |
| L-Leucine | 131.0 | 125.0 | 0.9541985 |
| L-Lysine | 146.0 | 75.0 | 0.51369864 |
| L-Methionine | 149.0 | 75.0 | 0.5033557 |
| L-Phenylalanine | 165.0 | 125.0 | 0.75757575 |
| L-Serine | 105.0 | 200.0 | 1.9047619 |
| L-Threonine | 119.0 | 300.0 | 2.5210085 |
| L-Tryptophan | 204.0 | 20.0 | 0.09803922 |
| L-Tyrosine | 181.0 | 300.0 | 1.6574585 |
| L-Valine | 117.0 | 100.0 | 0.85470086 |
| **Vitamins** | | | |
| Choline chloride | 140.0 | 1.0 | 0.007142857 |
| D-Calcium pantothenate | 477.0 | 1.0 | 0.002096436 |
| Folic Acid | 441.0 | 1.0 | 0.0022675737 |
| Niacinamide | 122.0 | 1.0 | 0.008196721 |
| Pyridoxine hydrochloride | 206.0 | 1.0 | 0.004854369 |
| Riboflavin 5'-phosphate Na | 478.0 | 0.1 | 2.0920503E-4 |
| Thiamine monophosphate | 442.0 | 1.0 | 0.0022624435 |
| i-Inositol | 180.0 | 2.0 | 0.011111111 |
| **Inorganic Salts** | | | |
| Calcium Chloride (CaCl2) (anhyd.) | 111.0 | 140.0 | 1.2612612 |
| Magnesium Chloride (anhydrous) | 95.0 | 93.7 | 0.9863158 |
| Magnesium Sulfate (MgSO4) (anhyd.) | 120.0 | 97.67 | 0.8139166 |
| Potassium Chloride (KCl) | 75.0 | 400.0 | 5.3333335 |
| Potassium Phosphate monobasic (KH2PO4) | 136.0 | 60.0 | 0.44117647 |
| Sodium Chloride (NaCl) | 58.0 | 8000.0 | 137.93103 |
| Sodium Phosphate dibasic (Na2HPO4) anhydrous | 142.0 | 190.0 | 1.3380282 |
| **Other Components** | | | |
| D+ Galactose | 180.0 | 900.0 | 5.0 |
| Sodium Pyruvate | 110.0 | 550.0 | 5.0 |

Table S2. Advanced MEM.

| **Components** | **Molecular Weight (g/mol)** | **Concentration (mg/L)** | **Concentration (mM)** |
| --- | --- | --- | --- |
| **Amino Acids** | | | |
| Glycine | 75.0 | 7.5 | 0.1 |
| L-Alanine | 89.0 | 8.9 | 0.099999994 |
| L-Arginine hydrochloride | 211.0 | 126.0 | 0.5971564 |
| L-Asparagine | 132.0 | 13.2 | 0.1 |
| L-Aspartic acid | 133.0 | 13.3 | 0.1 |
| L-Cystine 2HCl | 313.0 | 31.0 | 0.09904154 |
| L-Glutamic Acid | 147.0 | 14.7 | 0.1 |
| L-Histidine hydrochloride-H2O | 210.0 | 42.0 | 0.2 |
| L-Isoleucine | 131.0 | 52.0 | 0.39694658 |
| L-Leucine | 131.0 | 52.0 | 0.39694658 |
| L-Lysine hydrochloride | 183.0 | 72.5 | 0.39617488 |
| L-Methionine | 149.0 | 15.0 | 0.10067114 |
| L-Phenylalanine | 165.0 | 32.0 | 0.19393939 |
| L-Proline | 115.0 | 11.5 | 0.1 |
| L-Serine | 105.0 | 10.5 | 0.1 |
| L-Threonine | 119.0 | 48.0 | 0.40336135 |
| L-Tryptophan | 204.0 | 10.0 | 0.04901961 |
| L-Tyrosine disodium salt dihydrate | 261.0 | 52.0 | 0.19923371 |
| L-Valine | 117.0 | 46.0 | 0.3931624 |
| **Vitamins** | | | |
| Ascorbic Acid phosphate | 290.0 | 2.5 | 0.00862069 |
| Choline chloride | 140.0 | 1.0 | 0.007142857 |
| D-Calcium pantothenate | 477.0 | 1.0 | 0.002096436 |
| Folic Acid | 441.0 | 1.0 | 0.0022675737 |
| Niacinamide | 122.0 | 1.0 | 0.008196721 |
| Pyridoxine hydrochloride | 204.0 | 1.0 | 0.004901961 |
| Riboflavin | 376.0 | 0.1 | 2.6595744E-4 |
| Thiamine hydrochloride | 337.0 | 1.0 | 0.002967359 |
| i-Inositol | 180.0 | 2.0 | 0.011111111 |
| **Inorganic Salts** | | | |
| Calcium Chloride (CaCl2) (anhyd.) | 111.0 | 200.0 | 1.8018018 |
| Magnesium Sulfate (MgSO4) (anhyd.) | 120.0 | 97.67 | 0.8139166 |
| Potassium Chloride (KCl) | 75.0 | 400.0 | 5.3333335 |
| Sodium Bicarbonate (NaHCO3) | 84.0 | 2200.0 | 26.190475 |
| Sodium Chloride (NaCl) | 58.0 | 6800.0 | 117.24138 |
| Sodium Phosphate dibasic (Na2HPO4-H2O) | 138.0 | 140.0 | 1.0144928 |
| **Proteins** | | | |
| AlbuMAX® II |  | 400.0 | Infinity |
| Human Transferrin (Holo) |  | 7.5 | Infinity |
| Insulin Recombinant Full Chain | 5964.0 | 10.0 | 0.0016767271 |
| **Trace Elements** | | | |
| Ammonium Metavanadate | 117.0 | 3.0E-4 | 2.5641027E-6 |
| Cupric Sulfate | 250.0 | 0.00125 | 5.0E-6 |
| Manganous Chloride | 198.0 | 5.0 | 0.025252525 |
| Sodium Selenite | 173.0 | 0.005 | 2.8901733E-5 |
| **Other Components** | | | |
| D-Glucose (Dextrose) | 180.0 | 1000.0 | 5.5555553 |
| Ethanolamine | 98.0 | 1.9 | 0.019387756 |
| Glutathione (reduced) | 307.0 | 1.0 | 0.0032573289 |
| Phenol Red | 37604.0 | 10.0 | 2.6592915E-4 |
| Sodium Pyruvate | 110.0 | 110.0 | 1.0 |

Supplementary 2. Influence of an increased membrane permeability

The membrane permeability can be influenced by the occurrence of aquaporin channels which act as passive transmembrane water transport system in many cells. In this case, the permeability coefficient *l*_m_ is regarded as the sum the permeability coefficients of the lipid bilayer (*l*_b_) and the water permeability through aquaporins (*l*_a_), *l*_m_ = *l*_b_ + *l*_a_. The predicted behavior at an increased membrane permeability is shown in Fig. S1.


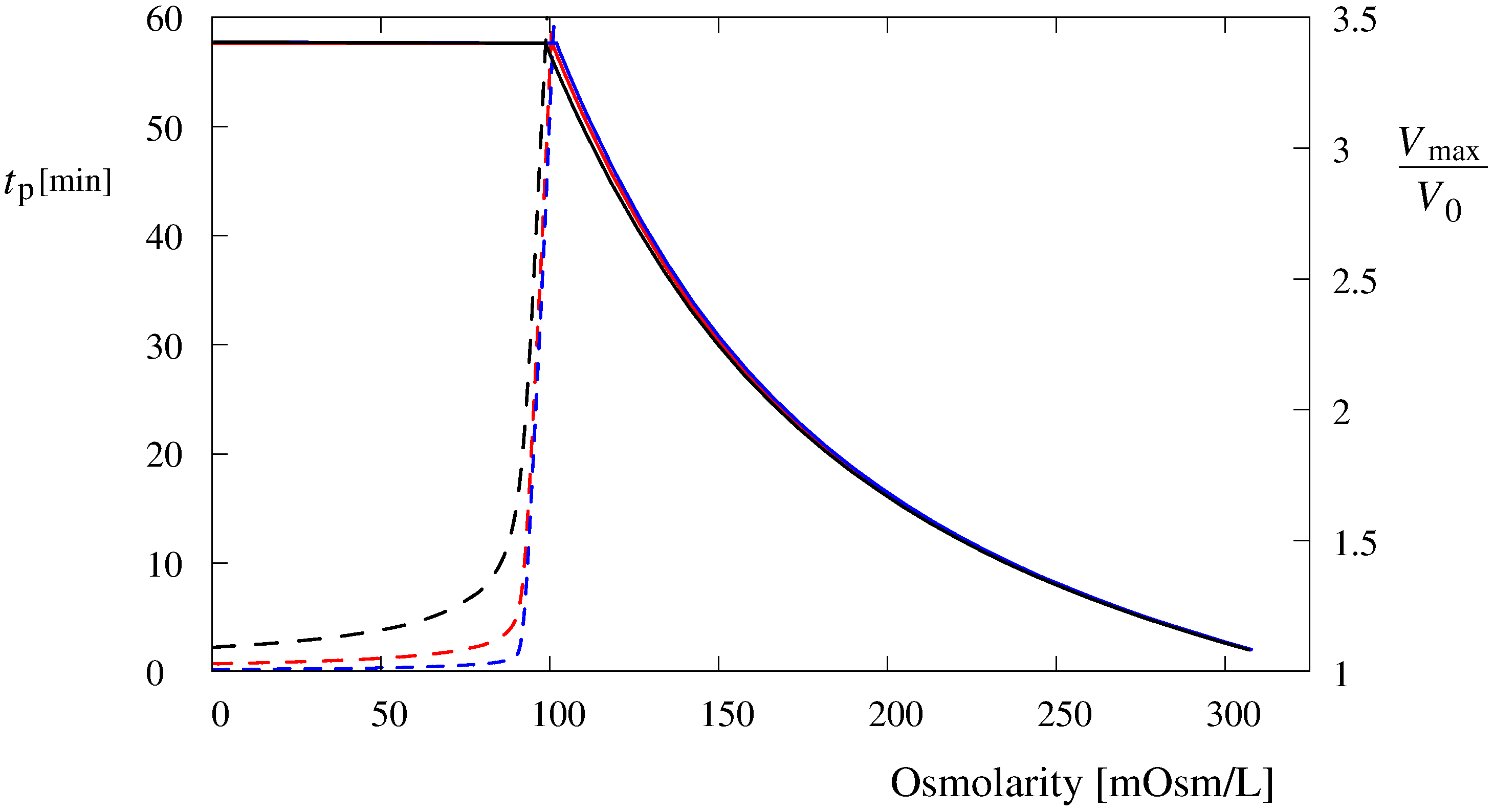


**Fig S1.** **Predicted occurrence time (*t*_p_) of the first tension pore (dashed lines) and maximum cell volume (*V*_max_/*V*_0_) (full lines) as a function of the osmolarity of Leibovitz-water** **solutions.** They are depicted for normal permeability (*l*_m_ = 9 × 10^-14^ m^3^/(Ns), black) as well as for 3 (red) and 10 (blue) times increased value of membrane permeability. The maximum cell volume is plotted relative to its initial value.
